# Microbiota is structured by gut regions, life stage, and diet in the Black Soldier Fly (*Hermetia illucens*)

**DOI:** 10.3389/fmicb.2023.1221728

**Published:** 2023-08-17

**Authors:** Laurence Auger, Marie-Hélène Deschamps, Grant Vandenberg, Nicolas Derome

**Affiliations:** ^1^Laboratory Derome, Département de Biologie, Institute of Integrative Biology and Systems, Université Laval, Québec, QC, Canada; ^2^Département des Sciences Animales, Université Laval, Québec, QC, Canada

**Keywords:** *Hermetia illucens*, microbiota, gut microbes, interactions, adaptative dysbiosis

## Abstract

The larvae of the Black Soldier Fly (*Hermetia illucens*) provide numerous ecological benefits, leading to significant commercial advancements. These benefits include the bioconversion of low-value waste into high-value feed and soil amendments. Understanding how the bacterial and eukaryotic microbiota communities affect host performance becomes vital for the optimization and specialization of industrial-scale rearing. This study investigates *H. illucens*-associated microbiota taxonomic composition and dynamics across the developmental cycle (eggs, neonates, larvae, prepupae, and imago X0 to second generation X1) when reared on two substrates: (i) plant-based (Housefly Gainesville diet) and (ii) animal-based (poultry hatchery waste). By using the 16S gene amplicon metataxonomic approach, we found that the results revealed that bacterial microbiota inherited from parents reared on a different substrate may have induced dysbiosis in the progeny. Specifically, the interaction networks of individuals reared on hatchery waste showed a high prevalence of negative interactions and low connectivity. Proteobacteria (39–92%), Firmicutes (4–39%), Bacteroidota (1–38%), and Actinobacteria (1–33%). In animal feed-reared individuals, Firmicutes reached the highest relative abundance (10–80%), followed by Proteobacteria (6–55%), Actinobacteria (1–31%), and Bacteroidota (0–22%). The rearing substrate was the main driver of microbiota composition, while the developmental stage influenced only the whole individual's bacterial microbiota composition. Gut regions were associated with distinct bacterial composition and richness, with diversity decreasing along the digestive tract. For the first time, microeukaryotes of the microbiota other than Fungi were investigated using 18S genetic marker amplicon sequencing with novel blocking primers specific to the Black Soldier Fly. Microeukaryotes are a neglected part of multitrophic microbiota communities that can have similar effects on their hosts as bacterial microbiota. Microeukaryotes from seven orders were identified in black soldier flies, including potential pathogens (e.g., Aplicomplexa group). Nucletmycea were the dominant class throughout development, followed by Holozoa and Stramenophiles. The eukaryote microbiota was structured by developmental stages but not by gut regions. Insights from this study are a stepping stone toward the microbiological optimization of black soldier flies for industrial rearing, highlighting how a synthetic microbiota assembly should be tailored to the rearing environment of the larvae at a targeted developmental stage.

## 1. Introduction

The Black Soldier Fly (BSF), *Hermetia illucens*, is a polyphagous and saprophagous insect used industrially to bioconvert organic waste into insect biomass for sustainable animal feed and, to a lesser extent, for human food (Lu et al., 2022). Frass is a major by-product of BSF rearing and is used as a biofertilizer that has the potential to replace chemical fertilizers (Klammsteiner et al., 2020a). Implantation of BSF rearing on organic residues from the agri-food sector for animal feed and frass for soil amendment promotes a circular economy that revalorizes waste and reduces the environmental impact of animal production while minimizing waste (Rehman et al., 2023). BSF also has the potential to provide us with new antimicrobial peptides in the fight against antibiotic resistance (Moretta et al., 2020).

The holobiont concept highlights how the functional ability, development, and health aspects of animals are the sum of the combined expression of the animal and its microbiota (Simon et al., 2019). The microbiota plays major roles in multiple aspects of the host, one of which is the facilitation of host nutrient intake (Read and Holmes, 2017). More particularly, insect microbiota commonly provide basic life functions and make resources and habitats available (Douglas, 2015). The composition of microbiota communities is a dynamic process. Pioneering communities are inherited from parents and recruited from the local environment in an ecological succession throughout the developmental stages that facilitate environmental adaptation, a process termed microbiota ontogeny. This is particularly important for BSF, which are polyphagous insects that must adapt to shifts in substrate from one generation to the next. Therefore, BSF is best approached under the holobiont paradigm, encompassing the BSF and its microbiota in their dynamic interactions, to better understand and improve the factors that control the bioconversion ability of the BSF.

The importance of the holobiont on BSF performance has led to investigations into the abiotic factors that modulate the microbiota. The rearing substrate (larval diet) is the most studied and well-known abiotic factor structuring the microbiota communities associated with BSF (Engel and Moran, 2013; Bruno et al., 2019; IJdema et al., 2022). BSF is also known to induce changes in the environmental microbiota through their development, in part through the production of frass (Cifuentes et al., 2020). In industrial rearing, frass represents more than half of the production. An added value of this biofertilizer over other commercially available products is the microbial soil amendment ability of frass (Klammsteiner et al., 2020a). In addition to providing key nutrients for plant growth, some frass microorganisms have the ability to inhibit the growth of plant pathogenic fungi through antibiosis (Arabzadeh et al., 2022). Therefore, characterization of how the BSF holobiont modulates the microbiota of the substrate throughout its transformation into frass is of essential importance for the industry's future. Only a few studies have previously investigated the microbial communities in the substrate alongside BSF microbiota, considering various factors such as initial substrate microbial communities, substrate type, developmental stage, and investigating either whole larvae, whole gut, or specific gut regions (Bruno et al., 2019; Jiang et al., 2019; Wynants et al., 2019; Cifuentes et al., 2020). The influence of the initial composition and their dynamics on substrate microbiota and dynamics on the whole larval and gut region microbiota during ontology has been neglected.

Other factors have also been found to modulate the composition and dynamics of the microbiota associated with BSF. This includes host genetics that contribute to driving gut microbiota diversity and composition in BSF (Khamis et al., 2020; Greenwood et al., 2021). The geographic location also influences the taxa present in the microbiota, most probably because the microorganisms available for recruitment tend to change with the location of the rearing facility (Wynants et al., 2019; Khamis et al., 2020). IJdema et al. (2022) reported that the age of the larva was found to account for a small portion of the bacterial microbiota variation. While the effect of the developmental stages on bacterial composition has been previously investigated, the study used a single-rearing substrate and obtained an unusually low number of taxa (2010 Amplicon Sequence Variants, ASVs) over four developmental stages (egg, larva, pupa, and imago; Querejeta et al., 2022). These experimental parameters did not allow the researchers to state whether the developmental stage as a deterministic factor of the bacterial microbiota's composition is substrate-dependent or not, as substrate was found to be a dominant factor of microbial composition.

The gut microbiota is of particular interest in host-microbiota research because of the microbiota's role in host nutrition. The larval BSF digestive tract is divided into three morphological and physiological regions conserved in insects, correlated with the sequential functional specialization of the digestive process (i.e., food ingestion, digestion, and nutrient absorption): the foregut, midgut, and hindgut (Bonelli et al., 2019). The BSF midgut is the longest part of the digestive tract, subdivided into three functional regions diverging in morphology and luminal pH values: the anterior midgut (pH 5.9), the middle midgut (pH 2.1), and the posterior midgut (pH 8.3; Bonelli et al., 2019). BSF midgut microbiota taxonomic composition varies between midgut regions, and the taxonomic richness and evenness are lower along the successive regions (Bruno et al., 2019). However, the foregut and hindgut have never been included in microbiological investigations of the digestive tract. Regional specialization of microbial communities in the midgut and the functional division of the digestive tract highlights the importance of accounting for the structural division of the gut microbiota along the digestive tract to identify the distinct communities of the gut and their relative contribution to the distinct digestive processes. In cellulose-digesting insects, the hindgut is a specialized region harboring bacteria and protozoa that digest cellulose (Nation, 2022).

It is also suggested that BSF harbors a cuticle microbiota, as a previous study found the gut microbiota communities had different taxa than those found on whole larvae (IJdema et al., 2022). BSF are holometabolous insects (i.e., undergo complete metamorphosis), a process by which they completely change their gut epithelial cells. Diptera are known to maintain a bacterial presence in the gut during metamorphosis. However, the BSF model has yet to be investigated (Bakula, 1969; Wong et al., 2011). Holometabolous insects undergo a significant reduction in the gut microbiota communities' absolute abundance during metamorphosis (Manthey et al., 2022). This is a known mechanism in insects to facilitate ecological niche shifts by eliminating some members of the microbiota (Hammer and Moran, 2019; Manthey et al., 2022). This shift in communities is a powerful adaptative tool and seems to be driven by the purge of gut content, followed by the upregulation of immune genes, translated into the secretion of immune effectors in the gut prior to pupation (i.e., lysozyme and AMPs; Johnston and Rolff, 2015; Johnston et al., 2019). This allows microorganisms from a new habitat to compete with established microbiota and colonize the gut, potentially facilitating the exploitation of new resources.

Another important contributing factor to shaping the progeny microbiota is the vertical transfer of parental microbiota. This colonizing route has not been specifically investigated for BSF. Interestingly, specific bacteria have been identified to mediate specific oviposition by BSF conspecifics, suggesting that some species are recruited independently of the rearing environment and may be inherited (Rehman et al., 2023).

Eukaryotic members of the microbiota could have a significant influence on their host. Parasitic protists of plants and animals influence the community dynamics of their hosts through biotic processes (Geisen et al., 2015; Worden et al., 2015; del Campo et al., 2020; Kodio et al., 2020). Similar to bacterial, archaeal, and fungal host–microbiota interactions, protists can provide accessible nutrients to their hosts, allowing them to exploit and adapt to different ecological niches. Unfortunately, the study of protists has been excluded from BSF microbial studies and is largely unexplored in host-microbiota systems (Laforest-Lapointe and Arrieta, 2018). This creates a vacuum of knowledge regarding important biotic components of ecological systems. This is in part due to the difficulties associated with the study of protists in host systems compared to that of bacteria and fungi microorganisms (see methodology).

The objective of this study was to investigate the composition, dynamics, and ontogeny of the bacterial, archaeal, and eukaryotic microbiota associated with the Black Soldier Fly (BSF). Specifically, we examined the changes in the microbiota across different developmental stages of BSF during a full life cycle. We compared the microbiota of BSF reared on two substrates (plant-based and animal-based) in both the whole larva and three gut regions (foregut + anterior midgut, middle midgut, and posterior midgut + hindgut). In addition, we explored, for the first time, the presence and taxonomic profile of eukaryotic microbiota other than fungi using BSF-specific gene sequencing. By conducting a comprehensive investigation of the substrate, developmental stages, and gut regions in a single experiment, the study aimed to determine the relative contribution of these factors to the ontogeny of the bacterial and archaeal microbiota in BSF. Furthermore, this study assessed the suitability of untreated chicken hatchery waste as a substrate for BSF rearing, considering its potential for a circular economy model. In addition, the study attempted to bridge the knowledge gap regarding non-fungal eukaryotes associated with BSF and their biodiversity. Overall, this research provides valuable insights into the microbiota of BSF and its dynamics, contributing to our understanding of the ecological interactions and functional roles of microorganisms in this economically important insect species.

## 2. Materials and methods

In this study, BSF was reared on two distinct substrates—plant-based and animal—to assess how the host-associated bacterial and eukaryotic microbiota's composition and dynamics were affected by (1) substrate, (2) developmental stage, (3) gut region, and (4) parental microbiota.

### 2.1. Black soldier flies

The first generation (X0) of black soldier flies sampled for the experiment originated from a colony reared at the *LAboratoire de Recherche en Sciences Environnementales et Médicales* (LARSEM) at Université Laval (Québec, Canada). The colony was re-established in 2020, with larvae continuously reared on the Gainesville substrate since (Hogsette, 1992). Flies are inbred to produce a homogenous population. The original colony, as well as all experiments described in this study, had the same constant environmental conditions: 30°C with 70% relative humidity and a photoperiod of 12:12 h light/darkness.

### 2.2. Experimental conditions

Two experimental substrates were used: (1) Gainesville substrate, the same used in the colony rearing (50% bran, 20% corn, and 30% alfalfa; Hogsette, 1992), and (2) hatchery waste substrate, provided by a local chicken hatchery (Couvoir Scott, Saint-Apollinaire, QC, Canada). This substrate is a homogenous mix of eggs, chicks, and other production residues ground into a fine paste (composition on an annual median basis: 35% protein, 21% lipid, 8% crude fiber, 9% carbohydrate, 27% ash, and a pH of 6.6; Dallaire-Lamontagne et al., 2022; Wageningen Academic Publishers, 2022). Both diets were stored at −20°C for at least 48 h and then thawed for 6 h before use. The water content of the substrates was adjusted to 70% humidity by adding a volume of distilled water according to the hatchery waste moisture content.

### 2.3. Experimental design and sampling

**Figure 1:**
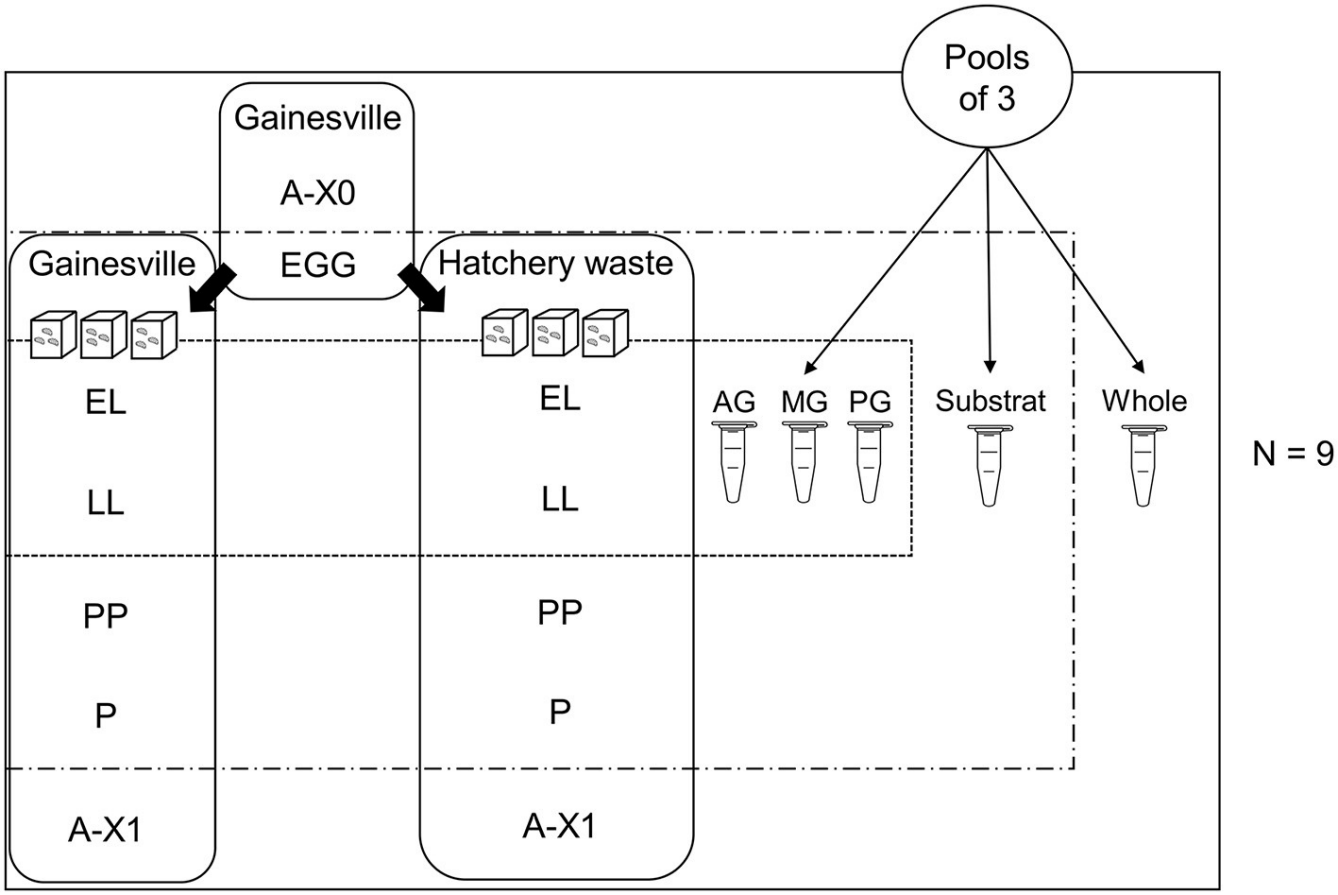
Experimental design. Visual representation of the experimental design, showing samples taken from (1) first generation imago (A-X0), (2) eggs laid by A-X0 (EGG), (3) neonates (EL, larvae day 4 post-hatching), (4) larvae (LL, day 8 post-hatching), (5) pre-pupae (PP, day 12 post-hatching, darkening of the cuticle), (6) pupae (P, day 21 post-hatching, dark cuticle and immobile), and (7) second generation imago (A-X1, day 35 post-hatching). Eggs were hatched in an empty sterile container before being transferred onto two substrates (plant-based Gainesville and animal-based hatchery waste, *N* = 9) to be reared until the end of the experiment.

An overview of the experimental design is presented in Figure 1. All eggs were collected at an interval of 8 h and allowed to hatch in sterile vented cell culture flasks (0.3 μm filter; Denville^®^) in the same abiotic conditions. Neonate larvae from all egg clutches were mixed; then, for each replicate, 300 larvae were transferred to a 1-L glass jar covered with fabric (Propper Steri-Wrap™, Thermo Fisher Scientific 11-890-8A) containing 400 g of substrate (nine replicates per substrate) within 12 h of hatching. Containers and fabric were previously sterilized individually in sterilizable bags by dry autoclaving for 30 min. Samples were a pool of three individuals randomly collected from each replicate of each rearing substrate (after thorough mixing with a sterile instrument) for each stage: (1) first generation imago (A-X0), (2) egg clutch (EGG; observed to have been laid by sampled parents A-X0), (3) neonates (EL; day 4 post-hatching), (4) larvae (LL; day 8 post-hatching), (5) pre-pupae (PP; day 12 post-hatching larvae with the darkening of the cuticle observed), (6) pupae (P; day 21 post-hatching, black cuticle and immobile), (7) second generation mature flies (A-X1). The substrate was sampled at EL, LL, PP, and P stages after thoroughly mixing with a sterile spoon.

Sampled flies A-X0 were observed laying eggs and were sampled after oviposition had ended, directly from the parent colony (reared in Gainesville). Eggs sampled and used in experiments were those previously observed to have been deposited in carboard egg-laying supports by the sampled A-X0 flies. Therefore, A-X0 and eggs were the only stages without replicates on hatchery waste substrate since they were sampled in the parent colony.

For each of the nine replicates of each substrate, the digestive tracts of the LL and PP stages were dissected and divided according to the regionalization (AG = anterior midgut including foregut, MG = middle midgut, PG = posterior midgut including the hindgut (pool of three by sample), as described by Bonelli et al. (2019). Sampled whole larvae were rinsed with abundant sterile water on a sieve to remove substrate adhering to the cuticle before being dried with UV-sterilized Kimwipes^TM^ (Kimtech Science^TM^). For gut sampling, the same previous steps were used, with a supplementary wash with 70% ethanol before drying. Then, dissection was performed under a flame. The whole gut was transferred to a new sterile dish before being divided into gut regions to prevent contamination by surface microorganisms. Before handling each new sample, scalpel blades were changed, and all instruments were sterilized using a flame.

### 2.4. DNA extraction

Before DNA extraction, samples were homogenized by crushing them with a sterile pestle. Genomic DNA extraction was performed with 50 mg of sample material (or the totality of the sample if the weight was <50 mg) by the salt extraction method described by Aljanabi and Martinez (1997), with an additional RNAase A step [4 μL at (10 mg/mL)] after the lysis step for 1 h at 37°C.

### 2.5. Bacterial library preparation

The V3-V4 regions of the 16S rRNA gene (500 pb) were amplified by PCR using the forward primer 341F: 5′ – ACACTCTTTCCCTACACGACGCTCTTCCGATCTCCTACGGGRSGCAGCAG – 3′ and the reverse primer 785R: 5′ – ACACTCTTTCCCTACACGACGCTCTTCCGATCTGACTACHVGGGTATCTAATCC – 3′ (Sylvain et al., 2020). All PCRs were performed according to the manufacturer's instructions for Q5 high-fidelity DNA polymerase (New England BioLabs, Inc.). The PCR amplification program was 1) 30 s DNA denaturation at 98°C, 2) 35 cycles of amplification steps of 10 s (denaturation) at 98°C, 30 s (annealing) at 64°C and 20 s (elongation) at 72°C, and a final elongation step, and 3) 2 min at 72°C.

### 2.6. Eukaryotic library preparation

The homolog of the 16S rRNA gene in Eukaryotes is the 18S rRNA gene. However, in the study of eukaryotic communities of host-associated microbiota, the host-specific 18S rRNA gene present in high abundance is simultaneously amplified, making other organisms' detection challenging. An efficient approach to bypassing this problem is the use of blocking primers (BPs) specific to the host that prevent the host target region from being amplified during library preparation (PCR). The blocking primer's function is to preferentially bind to the host DNA, and the presence of a C3 spacer at the 3′ end prevents enzymatic elongation of the primer, inhibiting the host. Peptide-nucleic acid (PNA) oligonucleotide blockers are used as elongation arrest BP, meaning BP that binds in between the two amplification primers has been found to have an efficiency of more than 80%, while anneal-inhibiting BPs (binding at the same site as PCR primers) were previously found to be much less effective (Belda et al., 2017).

Eukaryotic microbiota taxonomy sequencing and analysis were performed only on whole BSF and gut samples of BSF larvae reared on the Gainesville substrate. No environmental samples were collected as preliminary tests showed environmental (i.e., plants) DNA composed most of the sequencing reads, preventing the detection of eukaryotic microorganisms without developing an exhaustive repertoire of specific blocking primers, which was considered unrealistic. The 18S rRNA gene V9 hypervariable region was amplified from purified DNA. Amplification was performed using the forward primer 1389F 5′ – ACACTCTTTCCCTACACGACGCTCTTCCGATCTTTGTACACACCGCCC – 3′ and the reverse primer 1015R 5′ – ACACTCTTTCCCTACACGACGCTCTTCCGATCTCCTTCYGCAGGTTCACCT AC – 3′ (Amaral-Zettler et al., 2009). Host (*Hermetia illucens*) DNA amplification was reduced using the elongation arrest blocking primers method (Vestheim and Jarman, 2008). Candidate blocking primers were designed following the methodology described by Lundberg et al. (2013). Multi-sequence alignment comparison was done for the NCBI reference sequence of the *Hermetia illucens* 18S rRNA gene (AC: XR_005250514) against a selected eukaryote library from the RefSeq database with the MUSCLE alignment program implemented in UGENE to detect suitable unique sequences specific to the BSF for candidate blocking primer creation (Okonechnikov et al., 2012; O'Leary et al., 2016; Madeira et al., 2019; Sayers et al., 2022). The selected sequences had a length of at least 20 nucleotides in the V9 hypervariable region (not overlapping the selected primers) specific to the *Hermetia illucens* sequence, with at least three mismatching nucleotides with other eukaryotic sequences to prevent the blocking primers from binding to other eukaryotic 18S rRNA gene V9 sequences (Terahara et al., 2011). The blocking primers were further tested for their specificity *in silico* using the NCBI Primer-BLAST tool (Ye et al., 2012). Blocking primers were synthesized using Sigma-Aldrich^®^ with a C3 spacer (3 hydrocarbons, 1-dimethoxytrityloxypropanediol-3-succinoyl-long chain alkylamino), preventing the specific elongation of the *Hermetia illucens* V9 region of the 18S rRNA gene. The developed blocking primers were named BP-F_Hi_V9 (5′ – ATTTAGTGAGGTCTCCGGACGTG[SpcC3] – 3′) and BP-R_Hi_V9 (5′ – GGTCAACTTTTGCGAAACAACC[SpcC3] – 3′), blocking the elongation from the 1389F primer and the 1015R primer, and used at the same concentration and volume as the primers. The PCR amplification program was (1) 30 s denaturation at 98°C, (2) a cycle of 30 s (denaturation) at 98°C, 30 s (annealing) at 65°C, 1 min (elongation) at 72°C repeated 30 times, and (3) 10 min elongation at 72°C.

### 2.7. Sequencing

For both libraries, post-PCR DNA concentration and quality were assessed on Nanodrop, and by electrophoresis on 2% agarose gels, poor-quality samples were discarded. Amplified DNA was purified with magnetic beads (AMPure beads, Beckman Coulter Genomics^®^) and sequenced using the MiSeq platform from Illumina at *La plateforme génomique* at the *Institut de Biologie Intégrative et des Systèmes* (IBIS) at Université Laval (Québec, QC, Canada). A total of 305 samples were sequenced: 197 for bacterial 16S, including 71 whole (6 A-X0, 8 EG, 14 EL, 9 LL, 12 PP, 10 P, 12 A-X1), 76 gut samples (27 AG, 26 MG, and 23 PG), 43 environmental samples, and 105 for eukaryote 18S, with 48 whole (9 A-X0, 8 EG, 8 EL, 8 LL, 7 PP, 8 P, 8 A-X1) and 49 gut samples (18 AG, 14 MG, 17 PG); each sample constituted a pool of three individuals.

### 2.8. Sequencing data processing

All the data processing and analysis was conducted with R and Rstudio (R Development Core Team, 2010; RStudio Team, 2020). Reads were trimmed for quality using a phred score threshold of 20 and truncated to remove poor-quality bases at extremities (270 bp and 250 bp for forward and reverse 16S read cut-offs, respectively, and 160 bp for 18S reads). Short reads (under 250 bp) were discarded. An error prediction model was created using the DADA2 function for error rate learning, dereplication, and ASV inference (Callahan et al., 2016). Chimeric sequences were removed with the removeBimeraDenovo function of DADA2 (using the consensus method and a minFoldParentOverAbundance = 8), and ASV abundance tables were calculated. To prevent the potential presence of cross-contaminants in the dataset, ASVs identified in PCR negative controls were removed from samples using *decontam* with default values (Davis et al., 2018).

For bacterial analysis, chloroplast and mitochondrial reads were filtered out, and samples with fewer than 5,000 reads were discarded since the microbiota composition of these samples was considered unreliable (seven out of 197 samples). Contaminant ASVs were identified and removed using the *decontam* package (Davis et al., 2018). The final 190 samples had a total of 249,954 unique ASVs. The SILVA reference database (version 138.1) was used for the taxonomic assignment of bacterial ASVs (Glockner et al., 2017).

The following computations were conducted with the Phyloseq package (version 3.16; McMurdie and Holmes, 2013). Relative abundances' graphics were created with ggplot2 using a subset of 55,226 ASVs, as rare ASVs (<1 in at least 10% of samples) were removed to denoise the graphics (Wickham, 2016).

For eukaryote samples, Neoptera, Tetrapoda, and Chloroplastida reads were filtered out, and samples with fewer than 500 reads were discarded (eight samples, four EGG, and four A-X1). The final 97 samples had 705 unique ASVs, with a mean of 10,560 reads per sample. Taxonomic assignment of eukaryotic ASVs was conducted with the pr2-reference sequence database (version 4.14.0; Guillou et al., 2013; Vaulot et al., 2022).

### 2.9. Statistical analysis of the microbiota

Taxonomic communities' composition associated with BSF hosts was investigated to measure to what extent BSF microbiota ontogeny is influenced in terms of diversity and structure by the substrate, gut region, developmental stage, and generation. The dynamics of the microbiota's taxonomic composition in the rearing substrate were also monitored. The relative abundance of each taxon was measured and visually represented in stacked bar plots. Relative abundance was calculated using phyloseq at the Phylum and Class taxonomic ranks after filtering to keep only taxa present >1 in at least 10% of the samples (McMurdie and Holmes, 2015). Richness and evenness were measured with the alpha-diversity Chao1 index and the inverted Simpson and Shannon entropies (Chao and Shen, 2003). Statistical tests were performed with multivariate ANOVA (MANOVA), followed by Tukey High Significant Difference when normality was respected (confirmed by the Shapiro test) or by the Kruskal–Wallis test and the pairwise Wilcoxon test (when the normal distribution was rejected). In all statistical analyses, a significant threshold of α = 0.05 was used. Compositional diversity between sample groups was measured by clustering groups based on phylogenetic distance metrics (Weighted and Unweighted UniFrac) measured using the *vegdist* function of the VEGAN package (Dixon, 2003). The results were graphically plotted using non-metric multidimensional scaling (NMDS) ordination and principal component analysis (PCoA), and ellipses were drawn at a 95% confidence level (Lozupone et al., 2012). A subset of samples excluding the stages A-X0 and EGG was used when making statistical comparisons of BSF samples between substrates since these stages were from the parent colony. An analysis comparing BSF with their substrate samples was conducted with a subset excluding samples taken at the A-X0, EGG, and A-X1 stages since no substrate sample was taken for these stages. To quantify the strength of the association between investigated factors (rearing substrate, developmental stage, and gut region) and the sample group clustering, significance was measured using permutational multivariate analysis of variance (PERMANOVA) based on unweighted and weighted UniFrac distances with the *adonis* function, computed with 1,000 permutations. Since phylogenetic-based indexes need a phylogenetic tree to be calculated, a subset of the 5,000 most abundant taxa was used for the tests, the same subset used to build the phylogenetic tree (5,000 ASVs was found to be the upper limit for the phylogenetic tree calculations with our available resources). The results were verified using Bray–Curtis distance on the whole taxonomic dataset without filtering for the 5,000 most abundant taxa (see Supplementary material).

The core microbiota of BSF was analyzed for the entire sample set, regardless of the rearing substrate used. The detection threshold was set at 0.001 (1/1000), and three different prevalence thresholds were applied: 70% being the strictest, followed by 60 and 50% to define the core microbiota (Neu et al., 2021). The analysis was conducted at the ASV level.

To identify discriminant taxonomic groups whose relative abundance differed between sample types, linear discriminant analysis of effect size (LEfSe) tests were performed, with an LDA threshold score of 2.0 (default; Segata et al., 2011).

### 2.10. Interaction networks

Significant interactions in the microbial communities were investigated with co-occurrences/co-exclusion networks constructed using the CoNet tool (version 1.1.1 beta) plugin in the Cytoscape program (version 3.8.2), following the previously described approach (Shannon et al., 2003; Faust et al., 2012; Faust and Raes, 2016). The dataset used for these analyses was a subset excluding rare taxa (<1 in at least 10% of samples). Interactions were measured as per CoNet developer instructions, using Spearman correlations, Pearson correlations, Bray-Custis dissimilarity, and Kullback-Leibler dissimilarity distances based on co-abundance patterns of observed ASVs (Faust et al., 2012). The 100 top positive and negative interactions (edges) were selected. The compositional bias of the data was mitigated by computing final *p*-values with Brown's *p-*value merge method (100 iterations; Brown, 1975). Final *p-*values were adjusted with the Benjamini-Hochberg multiple testing correction (*p* < 0.05). Parameters were analyzed using the built-in Analyze application of Cytoscape as an undirected graph.

### 2.11. Data availability

All data are readily accessible from the Sequence Read Archive under the BioProject accession number PRJNA971599 in the NCBI BioProject database.

## 3. Results

After initial filtering steps, the dataset included 245,954 bacterial ASVs, with a mean of 37,368 ASVs and a median of 26,208 ASVs by sample (a minimum of 5,452 and a maximum of 190,353 ASVs). Rare taxa composed most of this dataset, as 190′728 ASVs were present in ≤ 1 read in 10% of the samples.

### 3.1. Bacterial microbiota composition in the environment

**Figure 2:**
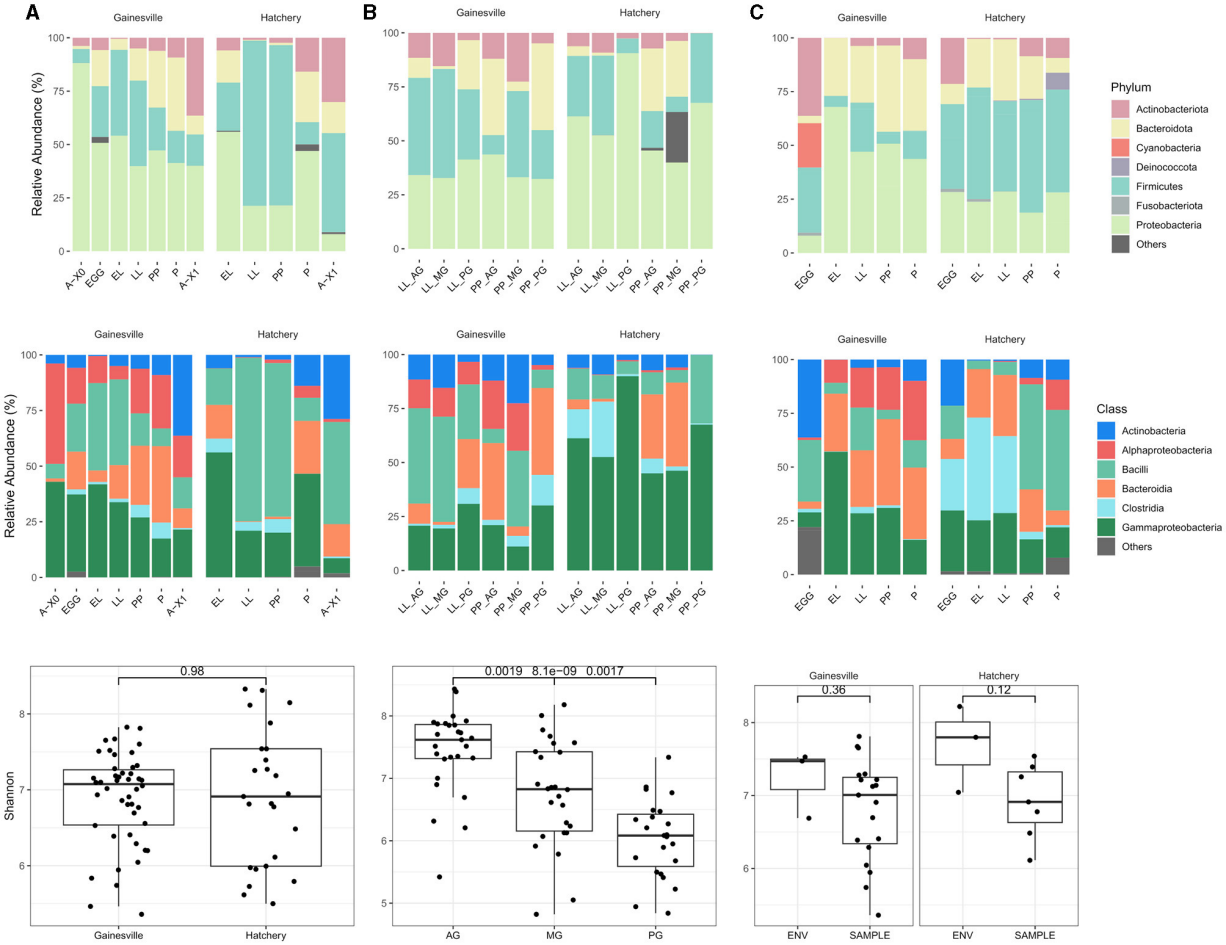
Bacterial microbiota composition and diversity. **(A)** Results for whole BSF samples by developmental stage (A-X0, first generation imago; EGG, eggs; EL, neonates; LL, larvae; PP, pre-pupae; P, pupae; A-X1, second generation imago), **(B)** results for gut region samples (AG, anterior gut + anterior, middle gut; MG, middle gut; PG, posterior middle gut and hindgut), and **(C)** for substrate samples. The first row presents the relative abundances (%) of bacterial phyla; the second row shows bacterial classes with a relative abundance of at least 5% (median). Taxa with a relative abundance <5% (median) have been grouped in the “others” category. The third row is a boxplot of the Shannon diversity index with comparative analysis of the alpha diversity for whole BSF samples reared in Gainesville or hatchery waste (first column), between gut regions (both substrates combined at both developmental stages sampled; second column) and between the substrate (ENV) and the whole BSF (SAMPLE) reared on the respective substrate (third column).

The most abundant bacterial taxa in all samples (i.e., whole BSF reared on Gainesville and on hatchery waste, gut regions from Gainesville and hatchery waste reared larvae, and environmental samples from the Gainesville and hatchery waste substrate) at the Phylum and class levels are shown in Figure 2. The relative abundance of major phyla across developmental stages of BSF reared on each substrate is also presented in Table 1, and the major phyla found in the gut regions are presented in Table 2. Before the introduction of BSF in the Gainesville substrate (control), the most dominant phyla in the substrate were Actinobacteria (36.3%), followed by Firmicutes (30.3%; Figure 2). Notably, the control Gainesville substrate was the only condition with a high abundance of Cyanobacteria (20.6%). Multiple phyla abundance decreased after the introduction of BSF (EL), including Actinobacteria and Cyanobacteria. During the same period, Proteobacteria, which initially had low abundance in Gainesville control, became and remained the most abundant phylum after BSF introduction. A similar trend was observed for the Bacteroidota phylum.

**Table 1:** Relative abundance of major phyla in whole BSF.

**Substrate**	**Stage**	**Proteobacteria**	**Firmicutes**	**Bacteroidota**	**Actinobacteria**
Gainesville	A-X0	92.2	4.7	1.0	2.0
	EG	48.9	25.0	21.6	3.7
	EL	55.1	39.2	5.1	0.7
	LL	39.9	38.0	19.0	3.2
	PP	48.0	22.0	24.6	5.4
	P	41.1	12.7	38.1	8.1
	A-X1	41.7	16.4	8.9	32.9
Hatchery	EL	55.2	22.4	15.8	6.0
	LL	18.3	80.4	0.4	0.9
	PP	27.8	68.5	1.1	2.5
	P	50.3	10.4	21.8	14.6
	A-X1	5.8	51.8	11.0	30.6

A-X0, first-generation imago; EG, egg clutch (laid by A-X0); EL, neonates (day 4 post-hatching); LL, Larvae (day 8 post-hatching); PP, Pre-pupae (day 12 post-hatching, darkening of the cuticle); P, Pupae (day 21 post-hatching, black cuticle and immobile); A-X1, second-generation imago.

**Table 2:** Relative abundance of major phyla in gut regions.

**Substrate**	**Stage**	**Region**	**Proteobacteria**	**Firmicutes**	**Bacteroidota**	**Actinobacteria**
Gainesville	LL	AG	34.1	45.0	9.3	11.6
		MG	32.8	50.5	1.3	15.4
		PG	41.3	32.5	22.7	3.4
	PP	AG	43.7	9.0	35.3	12.0
		MG	33.1	40.0	4.4	22.6
		PG	32.3	22.7	40.2	4.9
Hatchery	LL	AG	61.3	28.0	4.5	6.2
		MG	52.5	36.8	1.4	9.1
		PG	90.6	6.8	0.1	2.5
	PP	AG	45.5	17.0	29.0	7.2
		MG	39.9	7.1	25.8	3.7
		PG	67.6	32.2	<0.1	0.2

A-X0, first-generation imago; EG, egg clutch (laid by A-X0); EL, neonates (day 4 post-hatching); LL, Larvae (day 8 post-hatching); PP, Pre-pupae (day 12 post-hatching, darkening of the cuticle); P, Pupae (day 21 post-hatching, black cuticle and immobile); A-X1, second-generation imago.

### 3.2. Bacterial microbiota composition is associated with BSF

In both substrates, the most dominant phyla found in whole larvae were Proteobacteria, Firmicutes, Bacteroidota, and Actinobacteria (Figure 2; Table 1). In the BSF reared in Gainesville substrate, Proteobacteria was the main phylum with the highest relative abundance in A-X0 (92.2%), EG (48.9%), and all subsequent developmental stages. Proteobacteria classes found in BSF were Alphaproteobacteria and Gammaproteobacteria. Hatchery waste reared BSF also had Proteobacteria, however the most prevalent class was the Gammaproteobacteria.

In contrast to Gainesville-reared BSF, which exhibited a relatively stable distribution of phyla abundance across developmental stages, the BSF reared on hatchery waste substrate showed more variations. Firmicutes were the most abundant phylum at the LL (80.3%), PP (68.5%), and A-X0 (51.8%) stages, while Proteobacteria had the highest relative abundance only in the EL (55.2%) and P (50.3%) stages (Figure 2). Bacilli taxa had a high abundance in BSF at the EL and LL stages, the active feeding stages, on both substrates. In addition, Fusobacteriota, Deinococcota, Patescibacteria, Chloroflexi, and Desulfobacterota were found at diverse developmental stages in the hatchery waste substrate and in BSF reared on the same substrate. Desulfobacterota was the only persistent phylum in hatchery waste substrate in addition to the main phylum previously described.

Some phyla were only detected in BSF and not in the substrate, including Myxococcota (EG Gainesville), Bdellovibrionota (EG Gainesville; LL hatchery), and Gemmatimonadota (P, A-X1 hatchery).

Imago BSF bacterial microbiota shifted between A-X0 and A-X1 generations, even for BSF reared on the same substrate (Figure 2). Second-generation BSF showed increased Actinobacteria, Bacteroidota, and Firmicutes relative abundances for both substrates and a decrease in Proteobacteria.

The core bacteria present in at least 70% of the samples were two ASVs, respectively, the genus *Providencia* (Gammaproteobacteria) and an unidentified Lactobacillales (Bacilli). At a less stringent prevalence threshold of 60%, 20 ASVs constituted the core microbiota; seven were annotated to the *Providencia* genus, one to the *Morganella* genus, and 12 ASVs with an unidentified genus were Lactobacillales. Core microbiota at 50% prevalence included 69 ASVs: 6 *Morganella*, 39 Lactobacillales, 3 *Paenochrobactrum* (Alphaproteobacteria), and 20 *Providencia*.

### 3.3. Characterization of the BSF microbiota ontogeny

**Figure 3:**
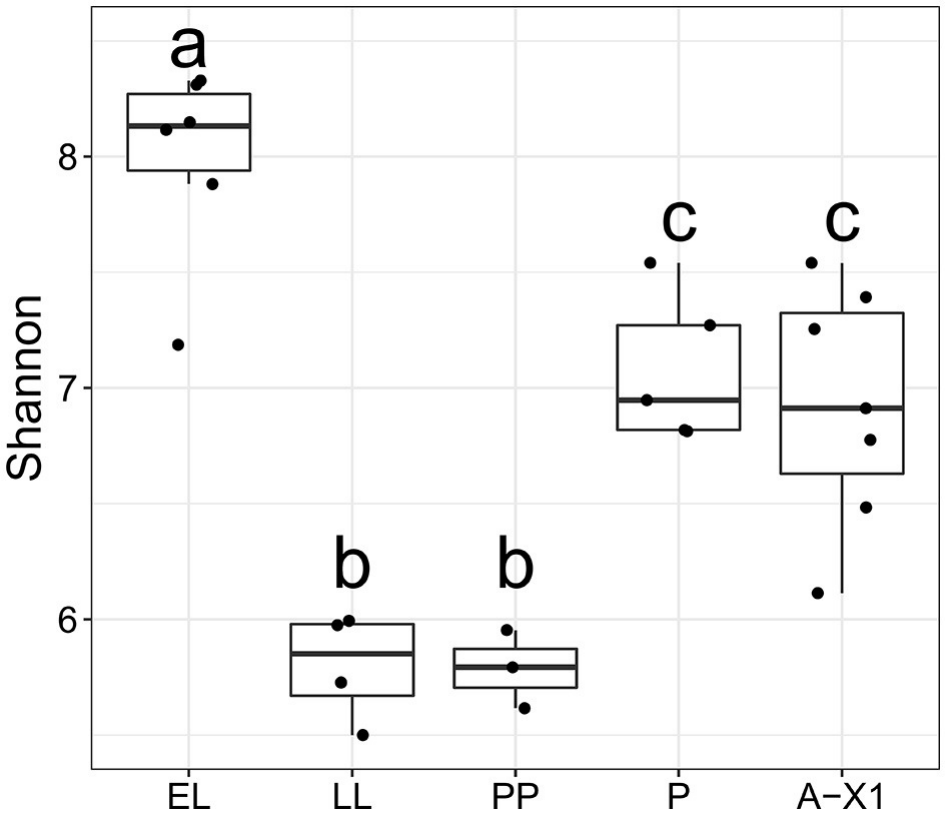
Bacterial diversity across developmental stages in BSF reared on hatchery waste. The α diversity (Shannon index) for whole BSF reared on hatchery waste at each developmental stage (A-X0, first generation imago; EGG, eggs; EL, neonates; LL, larvae; PP, pre-pupae; P, pupae; A-X1, second generation imago); a, b, and c indicate a significant difference between each letter group. The EL stage had the highest diversity of all stages (*p* < 0.05), while P and A-X1 both had the second highest observed diversity (*p* < 0.05). The LL and PP stages had the lowest diversity.

Rearing substrate had no impact on the BSF-associated bacterial microbiota community's species richness and evenness (α diversity), based on both Shannon entropy (*p* = 0.4975; Figure 2) and Chao1 species richness estimation *(p* = 0.5299). Alpha diversity also did not display differences between the two substrates' microbiota. Developmental stages did not affect BSF's bacterial α diversity reared on the Gainesville substrate *(p* = 0.9712, Shannon). However, α diversity did vary significantly (*p* < 0.05, Shannon; Figure 3) with the developmental stage of the BSF reared on hatchery waste. Diversity was highest at the EL stage, decreased for the rest of the feeding stages (LL and PP), and increased at the P and A-X1 stages but was still significantly lower than the EL stage diversity. The difference between the diversity of the whole BSF sample and their respective rearing substrates was also investigated using samples from all developmental stages. The Gainesville substrate bacterial community had higher α diversity than the BSF reared on it (*p* < 0.01), while hatchery waste-reared BSF showed no difference with their substrate. Species diversity was found to decrease along the digestive tract *(p* < 0.01, Shannon; Figure 2), from the highest diversity in the AG region to the lowest in the PG region. This trend was consistent regardless of the life stage or the rearing substrate.

**Figure 4:**
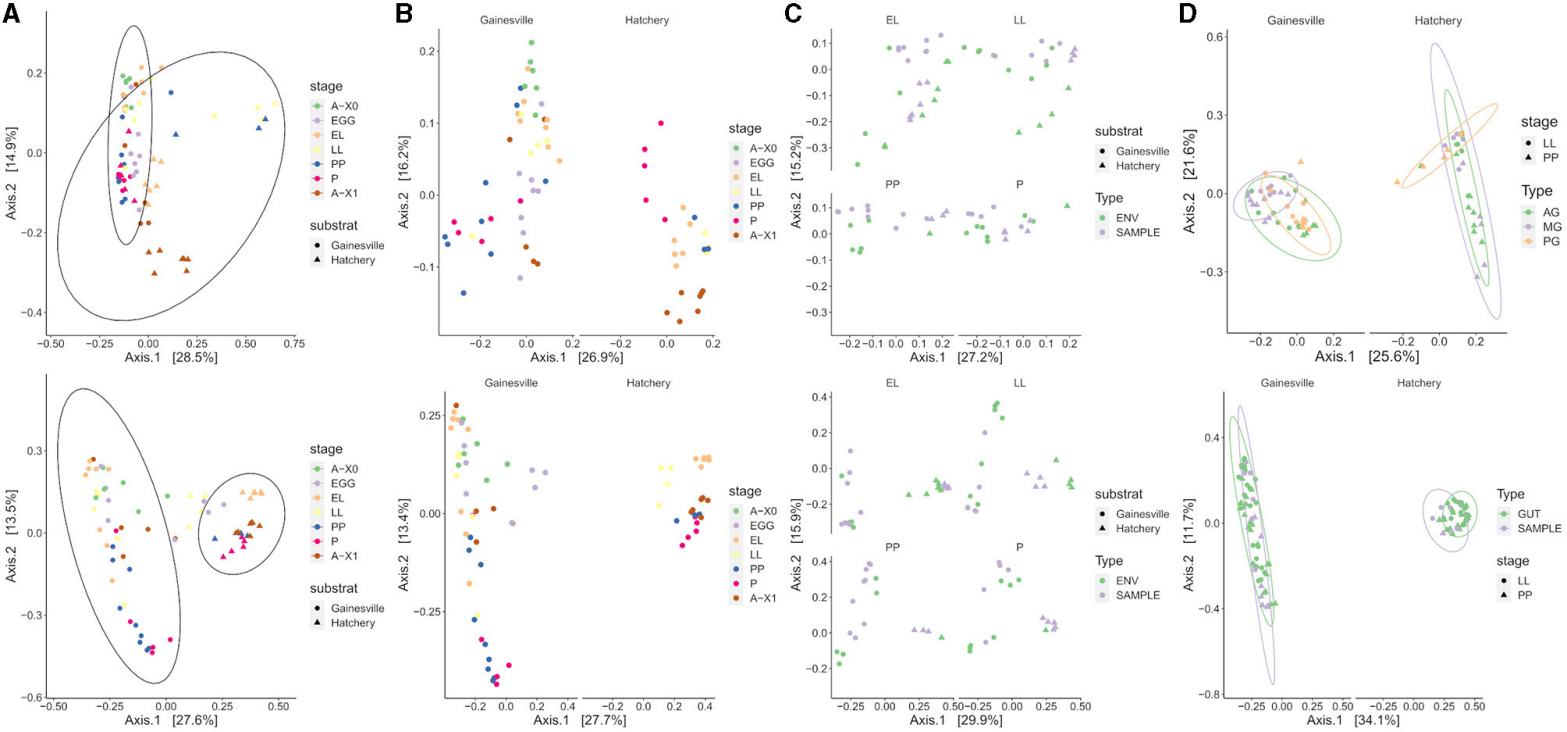
Clustering of the microbiota in ordination plots. The bacterial composition is clustered in PCoA ordination plots by weighted UniFrac (first row) and unweighted UniFrac distances (second row). **(A)** Whole BSF clustered significantly by their rearing substrate (PERMANOVA *p* < 0.01), **(B)** by developmental stage for both substrates (PERMANOVA *p* < 0.01), and **(C)** by sample type: substrate (ENV) or whole BSF (SAMPLE) for both substrates (PERMANOVA *p* < 0.01). **(D)** Gut regions clustered significantly by regions (AG, anterior gut + anterior, middle gut; MG, middle gut; PG, posterior middle gut and hindgut; PERMANOVA *p* < 0.01) for both substrates, but that the developmental stage had no effect based on weighted UniFrac (first row) and that the whole gut (all regions combined) did cluster significantly against the whole BSF (second row, PERMANOVA *p* < 0.01). A-X0, first generation imago; EGG, eggs; EL, neonates; LL, larvae; PP, pre-pupae; P, pupae; A-X1, second generation imago.

A graphical representation of BSF bacterial communities' beta diversity is presented in Figure 4. The rearing substrate was found to be the strongest factor explaining 27.6% of the variance in BSF bacterial microbiota (Unweighted UniFrac). Developmental stages explained 13.6% of the variance, while gut regions accounted for 10.1% of the variance. When considering the relative abundance of taxa (weighted UniFrac), the variance explained by the developmental stages (17.1%) and by the gut regions (19.8%) increased, while the variance explained by the rearing substrate decreased (23.6%) but remained the main explaining factor. Since these results were consistent across both unweighted and weighted metrics, this indicates the bacterial composition differed in terms of phylogenetic relatedness and evenness.

The taxonomic composition of the whole BSF samples' microbiota was significantly different than that of the rearing substrate for both conditions. The microbiota of the whole BSF was significantly structured by the developmental stage (*p* < 0.01). Variance explained 21.6 and 28.2% of the Gainesville and hatchery waste substrates, respectively (weighted UniFrac).

Gut regions also exhibited significant compositional differences at both the LL and PP stages. The stage did not significantly change the gut region's taxonomic composition. Interestingly, when combining the three gut regions to compare with the whole BSF larvae (pairwise comparison within the same stage at LL and PP), only BSF reared on hatchery waste did not share the same bacterial composition (*R*^2^ = 0.06521, *p* < 0.001).

**Figure 5:**
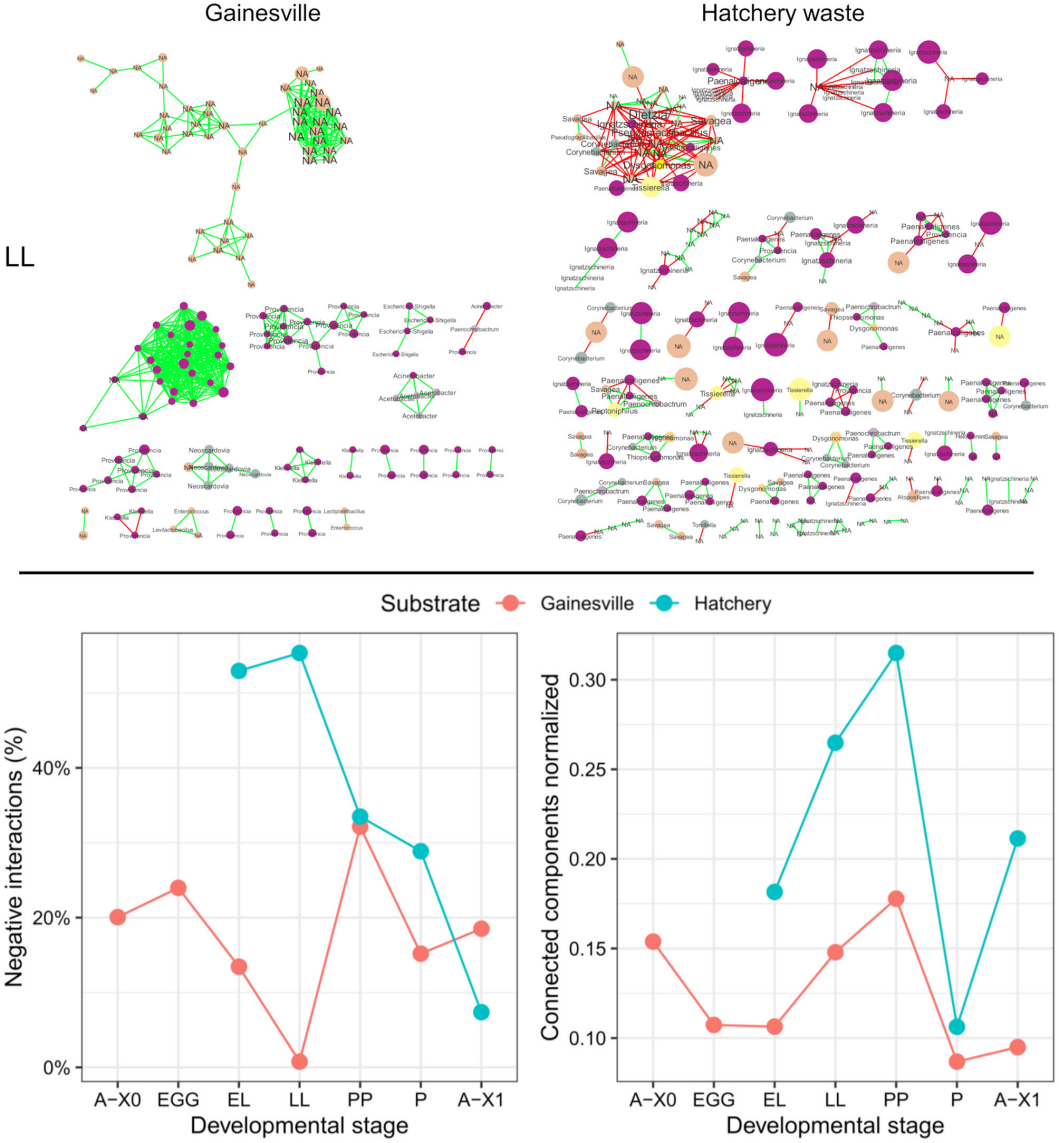
Interaction networks and parameters of the whole BSF microbiota. The first row shows the interaction networks of the microbiota at the LL stage for Gainesville **(left)** and hatchery waste **(right)** reared BSF. Edges represent significant interactions; red edges illustrate co-exclusion, while green edges indicate co-occurrence. Each node is a unique ASV, and node size is proportional to the relative abundance of each ASV in the sample group. Labels are the taxonomic family of the corresponding node, and font size is proportional to the number of connected edges (interactions). All the interaction networks are presented in the Supplementary material. The second row is a scatterplot resuming the evolution of interaction network parameters by developmental stages. The percentage of negative interactions (negative edges/total number of edges*100) is shown on the left, while normalized connected components (connected components/total number of nodes) is shown on the right (0 = all nodes are connected, 1 = no nodes are connected in the network). A-X0, first generation imago; EGG, eggs; EL, neonates; LL, larvae; PP, pre-pupae; P, pupae; A-X1, second generation imago.

The prevalence of negative interactions (in %) and the normalized number of connected components found in the networks of significant co-occurrence and co-exclusion relationships between the BSF microbial taxa at different developmental stages are presented in Figure 5. The networks of the BSF reared on Hatchery waste had a higher proportion of negative interactions at almost all developmental stages (EL = 53.0%, LL = 55.3%, PP = 33.5%, *P* = 28.9%) than in the Gainesville-reared BSF (EL = 13.5%, LL = 0.8%, PP = 32.1%, *P* = 15.2%), except for second-generation imagoes, which had a higher percentage of negative interactions in Gainesville (A-X1 = 18.5%) than in hatchery waste (A-X1 = 7.4%). Gainesville BSF interaction networks had stronger connectivity, with an average of 27 connected components consistently by stage compared to the average of 77 in hatchery waste BSF. The weakest normalized connectivity of BSF microbiota was found at the PP stage in both substrates, while the highest connectivity was at the pupal stage, which was also the stage with the highest number of significant interactions (see Supplementary material). Network heterogeneity was lowest at the P stage in both substrate BSFs (Gainesville = 0.279, hatchery waste = 0.556).

### 3.4. Discriminant features of the microbiota composition

**Figure 6:**
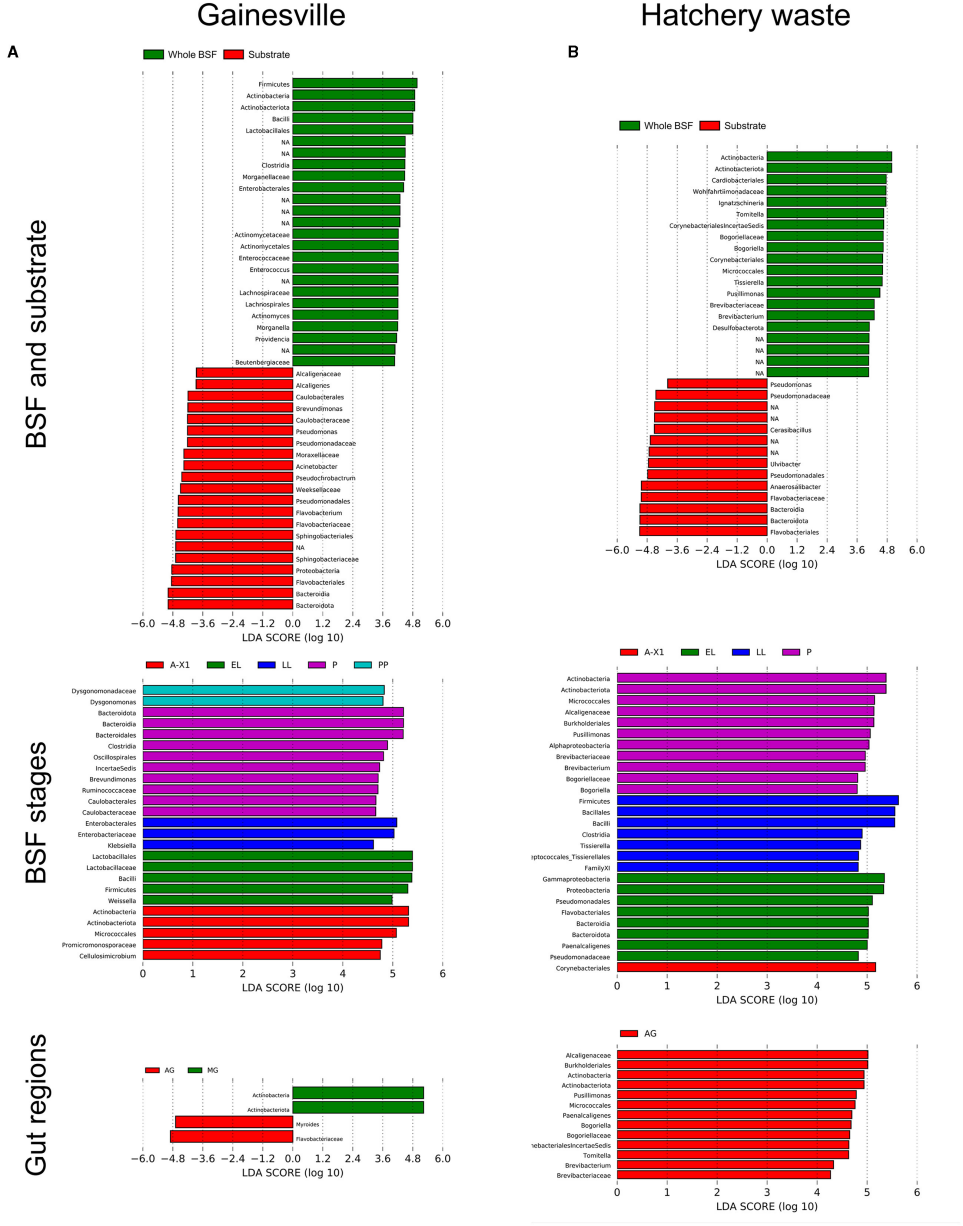
LEfSe analysis of the discriminant bacterial taxa between groups. LDA scores of the bacterial taxa are significantly discriminant (LDA > 4) between **(A)** Gainesville-reared BSF and **(B)** hatchery waste-reared BSF. Bacteria with a significant discriminant LDS score are shown: for whole BSF against their substrate (first row), for whole BSF by developmental stage (second row), and by gut regions (third row). A-X0, first generation imago; EGG, eggs; EL, neonates; LL, larvae; PP, pre-pupae; P, pupae; A-X1, second generation imago; AG, anterior gut + anterior middle gut; MG, middle gut; PG, posterior middle gut and hindgut.

The LDA scores for the LEfSe statistical analysis, identifying the taxa responsible for the compositional differences between groups, are shown in Figure 6. LEfSe results identified that in the Gainesville-reared BSF, the EL stage composition was characterized by Bacilli, the LL stage by Enterobacteriales, the PP stage by Dysgonomonas, the P stage by Bacteroidales, Clostridia, and Caulobacterales, and the A-X1 stage by Cellulosimicrobium. Regarding the gut regions, only the AG and MG had an increased abundance of some taxa when comparing all the regions simultaneously. In the AG, *Flavobacteriaceae* had a higher abundance, while in the MG, the class Actinobacteria had a greater abundance. The whole samples of BSF reared in Gainesville had compositional differences with their gut (all regions combined) samples (*R*^2^ = 0.03532, *p* < 0.001). Phylogenetic cladograms showing the discriminant taxa by taxonomic levels can be found in the Supplementary material.

When comparing whole BSF to their Gainesville substrate, BSF had a consistent difference in the relative abundance of the bacterial taxa: Actinobacteria (*Actinomyces*, Beutenbergiaceae, and unknown order), Bacilli (*Enterococcus* and unknown Lactobacillales), and Clostridia (*Lachnospirales*). The Gainesville substrate was characterized by a higher abundance of Bacteroidia (*Sphingobacteriales, Weeksellaceae*, and *Flavobacteriaceae*), Pseudomonales (*Pseudomonas* and *Acinetobacter*), Alcaligenaceae (*Alcaligenes*), and Caulobacterales (*Brevundimonas*). The Gainesville substrate composition changed after the introduction of BSF. In control Gainesville (EGG stage), the substrate was characterized by Actinobacteria (unassigned order), including Micrococcales. The substrate after the introduction of larvae was distinguished by the presence of the Gammaproteobacteria Enterobacteriaceae (*Klebsiella, Enterobacter*, and *Pseudocitrobacter*) and Brukholderiales, the Firmicutes *Lactobacillus*, and the Bacteroidia.

LEfSe results investigating the divergences in the relative abundance of the taxa associated with BSF when reared in Gainesville or on hatchery waste substrate across all developmental stages showed that the Gainesville BSF were characterized by more abundant Alphaproteobacteria (*Acetobacterales, Caulobacterales*, and *Rhizobiales*), Bacteroidia (*Bacteroidales* and *Sphingobacteriales*), *Dysgonomonas, Bifidobacteriaceae, Lactobacillales, Lachnospirales*, and *Oscillospirales*. In contrast, hatchery waste BSF had more Desulfobacterota, *Bacillales, Pseudomonadales, Cardiobacteriales, Peptostreptococcales*, and *Staphylococcales*. Interestingly, Gainesville BSF had two genera more abundant in the Micrococcales order (*Cellulosimicrobium* and an unknown Beutenbergiaceae), while hatchery BSF had two different genera more abundant in the same order (*Brevibacterium* and *Bogoriella*). For the Corynebacteria order, Gainesville BSF had *Corynebacterium* and *Dietzia* in higher abundance, while hatchery waste BSF had *Tomitella*.

BSF reared in hatcheries had a higher abundance of Flavobacteriales, Pseudomonadaceae, and *Paenalcaligenes* at the EL stage, Bacilliales and *Tissierella* at the LL stage, Alphaproteobacteria, *Bogoriella, Brevibacterium*, and *Pusillimonas* at the P stage, and Corymebacteriales at the A-X1 stage. The BSF reared on the hatchery when compared with their substrate, had a greater abundance of Desulfobacterota, Actinobacteria (*Tomitella, Brevibacterium*, and *Brogoriella*), *Ignatzschineria, Pusillimonas*, and *Tissierella*. In the digestive tract of BSF reared on hatchery waste, when comparing all the gut regions' bacterial composition at the same time, only the AG region had taxa whose abundance was specifically higher. Those taxa were Actinobacteria (*Brevibacterium, Bogoriella*, and *Tomitella*) and Burkholderiales (*Pusillimonas* and *Paenalcaligenes*). The hatchery substrate control (EGG stage) composition differed from the EL stage by the presence of Streptococcaceae, while the EL stage substrate was characterized by the presence of Desulfobacterota (unassigned class).

### 3.5. Eukaryotes in the microbiota

For the eukaryotic dataset, after the initial filtering steps, there were 77 samples left (Whole: 3 A-X0, 3 EGG, 8 EL, 8 LL, 7 PP, 4 P; Gut regions: 14 AG, 13 MG, 17 PG) with a total of 705 taxa. The dataset had a mean of 13,239 reads per sample, a median of 11,004, a minimum of 510 reads, and a maximum of 51,413 reads per sample.

**Figure 7:**
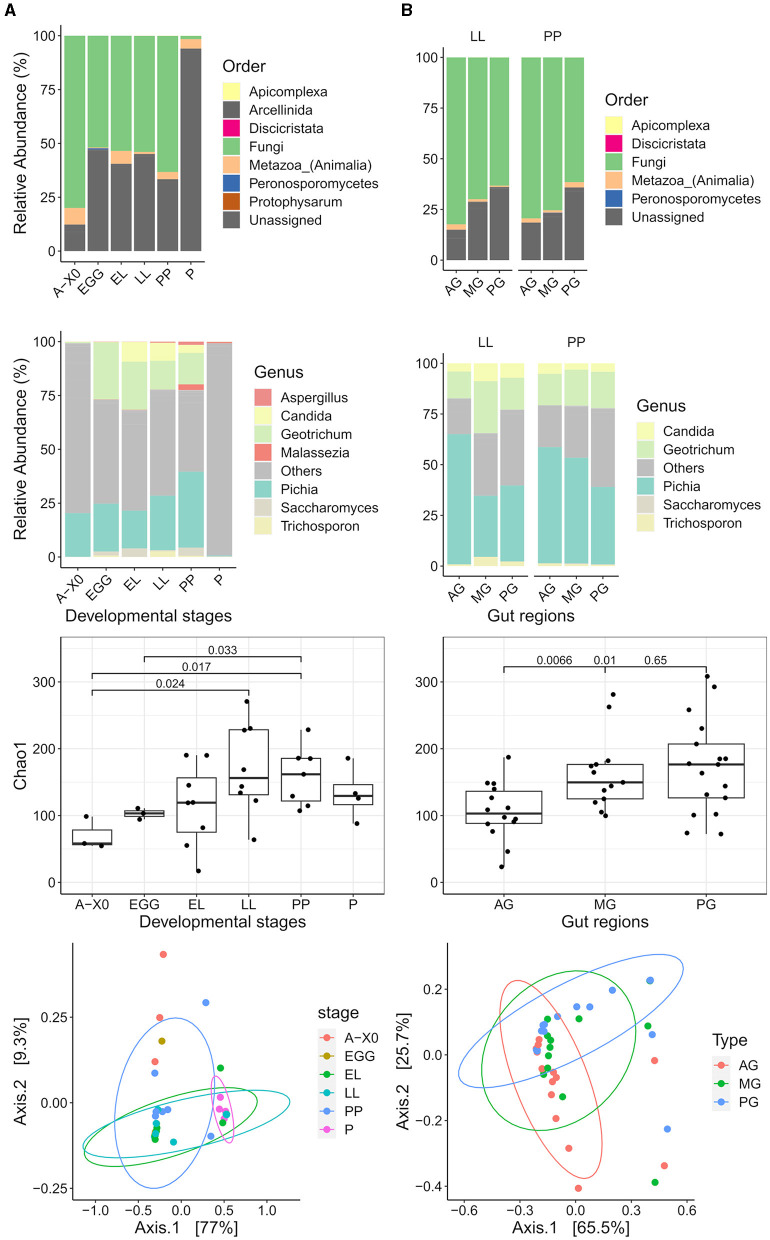
Eukaryotic microbiota composition, α diversity, and clustering. Relative abundance at the order taxonomic level (first row) and genus taxonomic level (second row) is presented for **(A)** the eukaryotic microbiota of the whole Gainesville BSF by developmental stages (A-X0, first generation imago; EGG, eggs; EL, neonates; LL, larvae; PP, pre-pupae; P, pupae; A-X1, second generation imago) and **(B)** the eukaryotic microbiota of gut regions (AG, anterior gut + anterior, middle gut; MG, middle gut; PG, posterior middle gut and hindgut). Unassigned taxa have been grouped in the order bar plots. In genus bar plots, unassigned taxa and taxa with a relative abundance <5% have been grouped in the “others” category. The differences observed for the α diversity (Chaos1 index) are presented (third row) with significant values (*p* < 0.05). In the whole BSF, diversity was significantly lower for A-X0 compared to the LL and PP stages. The PP stage diversity was also significantly higher than the EGG stage. In the gut regions, AG had the lowest diversity, significantly lower than both MG and PG. Clustering of the microbiota composition in PCoA ordination plots based on weighted UniFrac (row 4) distances show the eukaryote microbiota composition was significantly different for whole BSF samples across developmental stages (PERMANOVA *p* < 0.01) and between the gut regions (PERMANOVA *p* < 0.01).

The relative abundance of taxa at the order and genus levels is presented in Figure 7. There was a high abundance of ASVs that could not be assigned to a taxon starting from the class taxonomic rank. Both whole samples and gut regions were dominated by the clade Opisthokonta (>90% relative abundance). The supergroup SAR was the second most abundant (0.1–9%), mainly Stramenopiles (genus *Phytophthora*) at the EGG stage, then Apicomplexa (*Cryptosporidium* and Eugregarinorida) was the dominant SAR at the EL, PP, and P stages. The phyla Amoebozoa (Myxogastria and Tubulinea: Echinamoebida) and Excavata (Discoba: Tetramitia) were also detected (<0.1% relative abundance). Fungi were the dominant order in Opisthokonta (62–89%), except at the pupal stage, where Holozoa became most abundant (65%). However, these Holoza could not be annotated below the class level. A list of the eukaryote ASVs annotated at the species level can be found in the Supplementary material.

While ASV relative abundance varied between gut regions, detected community members were the same. Fungi were the dominant kingdom in each region (86% in AG, 80–93% in MG, and 74% in PG). Fungal species present were *Cyberlindnera fabianii, Diutina mesorugosa, Pichia kudriavzevii, Trichosporon asahii*, an uncultured *Geotrichum*, and an uncultured *Candida*. *Pichia kudriavzevii* had the highest abundance of all eukaryotes (30–64%), followed by an unidentified eukaryote. Other eukaryotes found were Apicomplexa (Eugregarinorida, *Cryptosporidium*), Arcellinida: Echinamoebida, Peronosporomycetes: Phytophtora, Discricristata:Percolozoa: Tetramitia, and Amoebozoa: Myxogastria.

Gut eukaryotic microbiota samples included all order taxa found in the whole sample except for the following fungal families: Ascodesmidaceae, Aspergillaceae, Aureobasidiaceae, Cuniculitremaceae, Debaryomycetaceae, Erysiphaceae, Malasseziaceae, Mucoraceae, Phaeosphareiaceae, Pleosporaceae, Rhizopodaceae, Saccharomycetaceae, Schizophyllaceae, and Sporidiobolacea.

Core eukaryotic microbiota members (present in at least 70% of samples) were composed of two *Pichia kudriavzevii* species.

### 3.6. The dynamism of the eukaryotic microbiota through ontology

**Figure 8:**
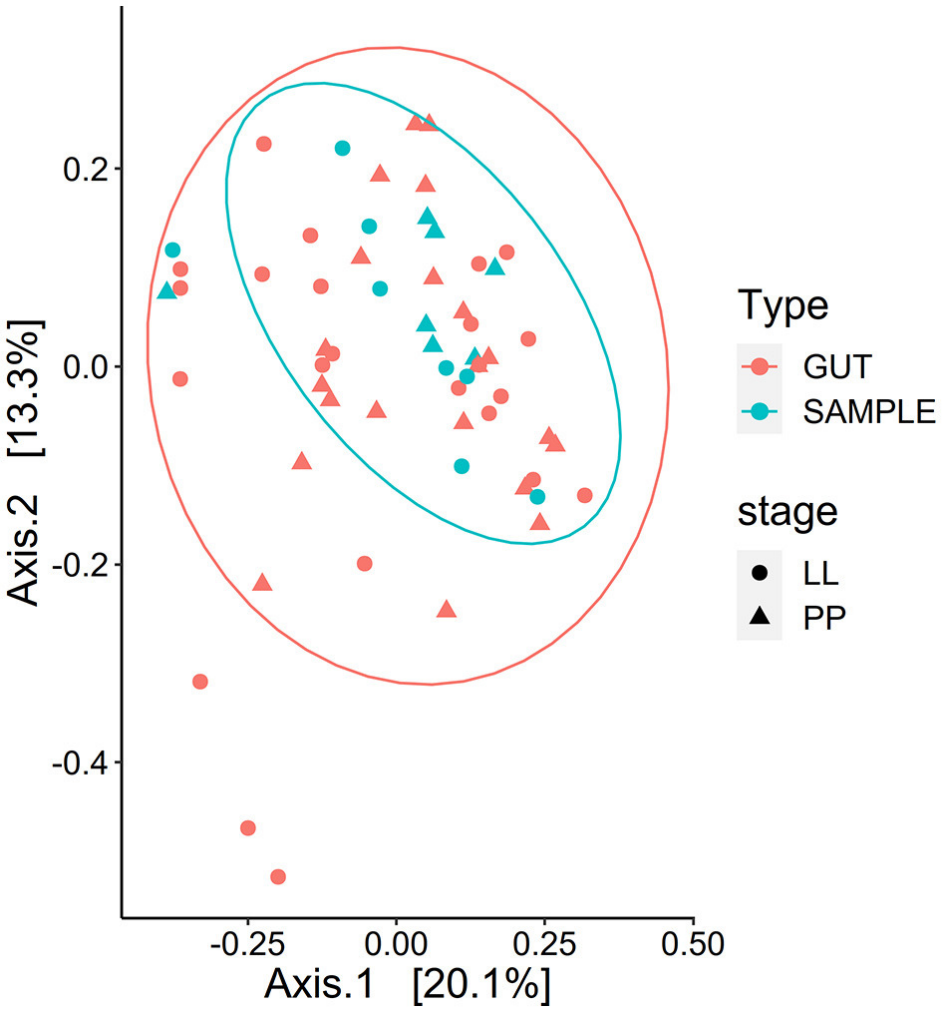
Clustering of the whole BSF and the gut regions combined. Clustering of the eukaryotic microbiota composition for whole BSF (SAMPLE) and whole gut (GUT, all regions combined) in the PCoA ordination plot based on weighted UniFrac distances. The whole larva had a marginally different composition than the gut samples (PERMANOVA *p* < 0.1).

Whole BSF and gut region microbiota composition clustering results for developmental stages and gut regions, as well as α diversity results, are presented in Figures 7, 8. Developmental stage had neither a significant effect on the evenness nor the richness of the eukaryotic microbiota in whole samples or gut samples. However, species richness based on the Chao1 index was structured by gut region (*F*-value = 5.536, *p* < 0.01) but not evenness, indicating rare eukaryotic taxa play a role in gut diversity.

The eukaryotic microbiota composition was structured by life stage (Weighted UniFrac *R*^2^ = 0.318, *p* < 0.01; unweighted UniFrac *R*^2^ = 0.260, *p* < 0.01). Gut eukaryotic microbiota composition clustered by regions based on unweighted distance (*p* < 0.01) but not when considering relative abundance (weighted UniFrac). However, gut samples (all regions combined) and whole BSF samples (pairwise at the same developmental stage) did significantly differ, as presented in Figure 8 (unweighted UniFrac *p* < 0.01 and weighted UniFrac *p* < 0.05. The developmental stage had no effect on the composition of the gut microbiota.

The LEfSe analysis did not find any discriminant taxa between developmental stages or gut regions.

## 4. Discussion

In the last few years, the rate of publications characterizing BSF microbiota has accelerated (Bruno et al., 2019; Wynants et al., 2019; Cifuentes et al., 2020; Khamis et al., 2020; Klammsteiner et al., 2020b; Gorrens et al., 2021; Tegtmeier et al., 2021; Zhang X. et al., 2021). However, comparison between studies is difficult, as many of these studies focus either on whole larvae, the whole guts, or on a single gut region: the middle gut. Furthermore, most studies focus only on the larval stage, overlooking the rearing substrate microbiota (environmental). Overall, there is little consensus across the previous studies on BSF microbiota taxonomy, even on the most abundant phylum observed. In addition, the relative contribution of factors structuring the microbiota is difficult to assess because they have not been examined simultaneously. In the current study, efforts were made to reduce individual sampling bias and be representative of the community: each condition and sampling time included three replicates, with each replicate being a pool of three individuals. While the larval stage is of particular interest for its importance in industrial uses, the factors structuring the microbiota in the other developmental stages have been neglected, even though they are essential aspects of BSF rearing. The use of 16S rDNA presents an important limitation as it does not allow for the identification of active taxa. However, this approach was chosen to avoid the loss of community diversity and spatial structure limitations associated with the 16S rRNA approach (Meyer et al., 2019).

This study also investigated the eukaryotic microbiota but was limited by two main factors. Overall, despite using specific BSF blocking primers, eukaryotic DNA from the host and other multicellular organisms (i.e., plants) dominated the sequence dataset, therefore limiting the detection power of microbial eukaryotes. Moreover, taxonomic annotation identified many potentially pathogenic organisms, which was to be expected due to the database bias toward pathogens. Nonetheless, new members of the eukaryotic microbiota have been identified, with enough consistency to speculate on members of a core eukaryotic microbiota. Fungal species were the most prevalent members of the eukaryotic microbiota. The structure of the eukaryotic microbiota followed similar trends to that of the bacterial microbiota.

### 4.1. Composition of the bacterial microbiota

In both rearing conditions, there were four main phyla: Actinobacteria, Bacteroidota, Firmicutes, and Proteobacteria (IJdema et al., 2022). This result was found to be generally consistent across studies, even if the relative abundance found varied. For BSF reared in Gainesville, Proteobacteria was the dominant phylum (39–92%) throughout all the successive developmental stages, followed by Firmicutes at the EG, EL, and LL stages, Bacteroidota at the PP and P stages, or Actinobacteria at the A-X1 stage (Tables 1, 2). These results highlight the high dynamism of the microbiota ontogeny in BSF.

The prevalence of Proteobacteria and lower abundance of Bacteroidota contrast with the results of previous studies using the same rearing substrate, Gainesville, where Bacteroidetes were the main associated phylum with larvae, and Proteobacteria were only the third most abundant phyla after Firmicutes (Bruno et al., 2019). The profiling of egg microbiota revealed the presence of microorganisms absent in the parental microbiota, likely transferred to the eggs from the cardboard used in the rearing facility as oviposition support. This means the facility's microbial community is another source of available microorganisms to be recruited by BSF larvae upon hatching and could contribute to the variation found between studies. Therefore, geolocation may determine microorganisms available for recruitment and may be a better predictor of the initial phyla's relative abundance found in substrate and BSF. This highlights the need for studies to report the initial microbiological composition of the rearing substrate before the introduction of BSF as a comparative baseline between studies.

### 4.2. BSF modulates the environmental microbiota

In this study, BSF changed the taxonomic composition and abundance of their rearing substrate microbiota. Gainesville substrate microbiota changed most significantly between the control substrate (EGG) and the substrate after the introduction of BSF (EL; Figure 2). In Gainesville, the four main phyla either increased in relative abundance after the introduction of BSF (i.e., Bacteroidota and Proteobacteria) or decreased (i.e., Actinobacteria and Firmicute). Comparatively, only the Actinobacteria phylum had a drastic change in abundance in hatchery waste. The BSF's ability to modulate the microbiota in the substrate has been reported previously (Bruno et al., 2019; Wynants et al., 2019; Cifuentes et al., 2020; Zhang X. et al., 2021). BSF can modulate environmental microbiota through the various antimicrobial peptides (AMPs) they produce, which are able to reduce the relative abundance of pathogens like *Salmonella* (Lopes et al., 2020; Elhag et al., 2022). However, contradictory results on the BSF's ability to reduce pathogen microbial loads suggest the BSF AMPs expression profile may be substrate-dependent as well (De Smet et al., 2021; Grisendi et al., 2022). BSF larvae also modify the physiochemical parameters of their feeding substrate, as evidenced in terms of pH changes, raising it to ~9.0 independently of the initial pH (Meneguz et al., 2018). These physiological modifications of the environment play a role in the modulation of the environmental microbiota. A germ-free study of BSF suggests that in the absence of microorganisms, the BSF expresses more genes related to xenobiotic biodegradation as time goes by on an unrefreshed substrate (Auger et al., 2023). Therefore, microorganisms play an important role not only in digestion and immunity but also in the detoxification of the environment, potentially by degrading waste produced by the larva that would otherwise accumulate in the environment, thus protecting the larva. The anti-biodegradation ability of the BSF seems to be, in great part, the result of their gut microbiota action (Cai et al., 2018).

### 4.3. Microbiota assembly and functions

The rearing substrate played a significant role in shaping BSF microbial assembly. The microbial composition of BSF was found to be closer to their respective environment's bacterial composition than the microbiota of the larvae reared on another substrate (Figure 4). The microbiota of second-generation imagoes (A-X1) was also significantly modulated by the larvae's rearing substrate. However, the results indicated that transmission from parents also plays a role in microbiota assembly, as microorganisms that were absent in the control substrate but present in eggs were also found in later developmental stages. While the substrate was an important modulator of the microbiota, the microbiota of BSF was still significantly different from their rearing substrate microbiota. Since population genetics are known drivers of the microbiota diversity in BSF, microbiota recruitment and assembly in BSF seem to be modulated by environmental microbiota, substrate type, parental microbiota, developmental stage, and genetic factors (Khamis et al., 2020; Greenwood et al., 2021).

Comparing the microbiota ontogeny of an entire reproductive cycle on two different rearing substrates provided further insights regarding the dynamic of dominant phyla from one generation to another. The α diversity of Gainesville-reared BSF's microbiota was relatively stable throughout the developmental stages: α diversity did not vary between developmental stages but was lower in the whole BSF than in the substrate. Interaction networks had relatively high interconnection (an average of 27 connected components by the developmental stage) and a low percentage of negative interactions (Figure 5). All these results indicated that the microbiota of larvae reared on the same substrate as their parents had a stable microbiota assembly with high colonization resistance (Vollaard and Clasener, 1994). Comparatively, the microbiota of BSF reared on hatchery waste had significant changes in α diversity across development and did not differ significantly between the whole BSF and the substrate. It also showed a significant difference in taxonomic composition between all gut regions combined and whole larvae, and the most dominant phyla in whole BSF changed at three separate developmental stages. Furthermore, interaction networks had consistently more separated modules than Gainesville (an average of 77 connected components by developmental stage) and a continuously greater prevalence of negative interactions than in Gainesville-reared BSF, except at the A-X1 stage. More than 50% of the significant interactions in the feeding stages of hatchery waste BSF were negative (EL = 53.0%, LL = 55.3%). This is of particular interest since a high prevalence of negative correlations in microbial interaction networks and low connectivity are indicators of dysbiosis (Vazquez-Baeza et al., 2016). These results showed low colonization resistance in the hatchery waste BSF microbiota. However, this may have been the result of an adaptative mechanism of the BSF to adapt to a new habitat. This adaptative mechanism has been suggested as a strategy for holometabolic insects like the BSF to renew their microbiota during metamorphosis since the total abundance of microbiota is reduced. This suggests the environmental microbiota will compete with the previous gut microbiota to establish itself after the change in environment. Therefore, the dysbiosis observed in hatchery waste BSF may be an adaptative dysbiosis necessary to allow the recruitment of a microbiota adapted to the environment, thus contributing to metagenomic plasticity (Alberdi et al., 2016; Boilard et al., 2020).

The first larval sample was taken 4 days after the environmental change for BSF reared on hatchery waste. BSF at this stage (EL) had the highest α diversity. This higher diversity may have arisen from the rearrangement of the microbial communities toward a configuration optimized for the new substrate (Marasco et al., 2022). Since eggs did not hatch in the substrate but rather in oviposition support, the neonate's digestive tracts were first colonized by the egg's microorganisms, including those left by the laying parent. The first microbiota recruited by neonates (from the egg surface) faces highly competitive interactions with the microorganisms of the substrate. This could explain the increased taxonomic diversity of the microbiota in EL reared on hatchery waste relative to Gainesville, as new microorganisms from the substrate were recruited and the community's assembly was rearranged. Conversely, EL reared in Gainesville showed virtually no change in richness since they inherited microbiota from parents reared on the same feeding substrate and were thus similar to and adapted to the Gainesville environmental microbiota, minimizing competitive interactions between egg surface microbiota and Gainesville substrate microbiota (Figure 3). This indicates that vertical transfer of key symbionts could be essential to ensuring quick adaptation to the rearing substrate. As previously mentioned, in the interaction networks (Figure 5), hatchery waste BSF microbiota showed clear signs of dysbiosis with a high number of negative interactions and relatively low connectivity. Therefore, competition for resources between BSF juvenile maladapted microbiota and the natural microbiota of the new substrate seemed to induce dysbiosis and could be a key factor explaining the low performance of BSF in nutrient-rich feeds. Since the microbiota did not stabilize in hatchery waste BSF, adaptation to a new substrate may take multiple generations for the BSF to assemble a stable microbiota optimized for the new habitat.

Second-generation imagoes had significantly different microbiota profiles than the first-generation on both substrates. Overall, the vertical transfer of microorganisms was limited from one generation to another since the microbiota composition of second-generation imagoes (A-X1) was significantly different from that of first-generation imagoes (A-X0) for both rearing substrates. The number of negative interactions in BSF microbiota also decreased in both substrates from the PP stage to the A-X1 stage, which was also the hatchery waste BSF stage with the lowest proportion of negative interactions (Gainesville: PP = 32.1%, *P* = 15.2%, A-X1 = 18.5%; hatchery waste: PP = 33.5%, *P* = 28.9%, A-X1 = 7.4%).

Since BSF are not social insects and are therefore expected to have limited horizontal transmission of symbionts, the holometabolous process may explain the low inheritability from one generation to another and the absence of consensus on which symbionts define the BSF core microbiota or known important symbionts that have co-evolved with this host, contrary to many other insect species. These results confirm the importance of the rearing substrate in driving host gut microbiota taxonomic composition and further stress that the rearing substrate compositional dynamic itself influences the dynamic of dominant phyla from one generation to another. The metagenomic plasticity of the microbiota in the polyphagous BSF could be an important trait for the rapid adaptation to a new feed substrate through the recruitment of a microbiota with functional competences that optimize the exploitation of the substrate (Alberdi et al., 2016).

These results suggest that BSF microbiota assembly adapts to the rearing substrate, favoring microorganisms that are functionally adapted to use the available nutrients (e.g., the ability to produce cellulase in vegetable substrates), reducing their complexity, and facilitating the bioconversion of these nutrients by BSF. This could be the case for Ruminococcaceae, a bacterial family that was initially absent in BSF in the A-X0 and EG stages but initially present in both rearing substrates. Ruminococcaceae was later observed in plant-based reared BSF at the EL stage. Ruminococcaceae can degrade cellulose and hemicellulose found in plant material (Chassard et al., 2012). This preferential assembly was also suggested by the discriminant bacteria observed. The discriminant bacteria that characterized BSF in one condition generally had functions that enabled the exploitation of that specific substrate (Figure 6). Bacteroidia (*Bacteroidales* and *Sphingobacteriales*) exhibited a distinct association with the Gainesville-reared BSF, as did *Dysgonomonas, Bifidobacteriaceae, Lactobacillales, Lachnospirales*, and *Oscillospirale*. *Dysgonomonas* are lignocellulose degraders prevalent in xylophagous insects that have been suggested to have a role in BSF polysaccharide catabolism (Bridges and Gage, 2021). *Bifidobacteriaceae* are glucose metabolizers that are regarded as probiotics (Ventura et al., 2004). *Lactobacillales* are carbohydrate fermenters found in vegetables and fruits (Hommel, 2014). The *Weissela* genus is associated with plants and fermented foods, with several strains being considered probiotics. *Lachnospirales* are fermenters of plant polysaccharides (Boutard et al., 2014). *Oscillospirales* are dependent on other bacteria or sugars generated from host mucins (Kohl et al., 2014).

### 4.4. Distinct microbiota between the larva's body and gut

There was no starvation step preceding the sampling of whole larvae or gut regions. Therefore, the presented results also included the transient microbiota (i.e., those associated with digesta) along with the mucosal microbiota. This may be more representative of the actual microbiota existing in association with the host during the rearing process, as BSF larvae are intermittent eaters (Shishkov et al., 2019). Investigating the gut microbiota without including the transient microbiota would prove challenging. The size of the digestive tract makes the mechanical removal of the transient gut content difficult, and starvation is known to perturb microbiota by reducing community structure and functions in BSF larvae (Yang et al., 2021).

In a pair-wise comparison by stage, the composition of the gut microbiota (all regions combined) was different from the whole BSF larvae microbiota. This difference in composition between the whole BSF and the gut supports the theory of a cuticle or insect body microbiota since no specialized organ hosting symbionts (i.e., a bacteriome) has been identified in BSF (IJdema et al., 2022).

### 4.5. The gut bacterial microbiota is differentiated across regions

The bacterial microbiota is known to decrease in observed taxa diversity along the structural and functional domains of the midgut (Bruno et al., 2019). The results of the current study confirmed this trend for the whole BSF digestive tract in terms of richness and evenness, regardless of the developmental stage and substrate. This was expected as the BSF digestive tract pH is known to change drastically between midgut regions; therefore, microorganisms must resist the selective pressure of the gut regions prior to reaching their colonization site (anterior midgut = 7 > pH ≥ 4.6; middle midgut pH ≤ 3; posterior midgut pH ≥ 8; Bonelli et al., 2019). Members of the microbial community in the PG must transit through and survive the MG's extremely acidic environment and then compete with other microorganisms to establish themselves in the PG's alkaline environment. Therefore, a lower diversity is expected to reflect the narrow selection of the available species in the environment.

The presented results revealed the axial distribution of bacteria along the digestive tract. This could be the result of the selective pressure exerted by the functional regions previously discussed. It would then be expected that the AG composition would be closer to the environmental microbiota, but the AG bacterial community composition was significantly different than that observed in the environment. At the PP stage, there was a sudden higher relative abundance of Desulfobacteria in the hatchery waste substrate, which was reflected in the MG region of the BSF but not in the other regions. This supports the hypothesis that MG is more strongly influenced by environmental microbiota than other gut regions (Bruno et al., 2019).

### 4.6. Core microbiota: should we even talk about it?

The characterization of the BSF's “core microbiota” has become widespread recently (Bruno et al., 2019; IJdema et al., 2022). The term core microbiota refers to a group of microorganisms that are closely associated with a specific host. This microorganism group can either be a set of taxa or a group of functional profiles that are shared among individuals of the host species and meant to represent the most evolutionary-relevant microbiota members or functions. However, the definition of core microbiota lacks consensus on its quantified definition: the occurrence and relative abundance thresholds of shared taxa used to identify core members of the microbiota (Neu et al., 2021). There is also no predetermined taxonomic level that must be used. More importantly, many factors such as sequencing depth, sampling time, tissue type, location, rearing substrate, and/or population may bias the inclusion of many taxa as core members, which may not be associated with the host organism at the species level but at the population level, for instance.

Therefore, the “core microbiota” in association with BSF in this study was deemed an experimental population core microbiota. The test was conducted using all the BSF samples from this experiment, meaning whole individuals and gut samples on both rearing substrates. The reported taxa had to be present in at least 70% of these samples. At the genus level, only *Providencia* and an unassigned *Lactobacillus* met these criteria. These results, combined with the high variability of the BSF microbiota, are indicative that the BSF core microbiota may be better represented by a set bacterial functional profile than a set of taxa.

### 4.7. Eukaryotic microbiota composition and dynamics

For the first time, the detection of eukaryotic microbiota species associated with the BSF other than fungal members was reported. This was made possible by the development in this study of PNA blocking primers specific to the BSF V9 region of the 18S rRNA gene that were able to significantly reduce the abundance of Neoptera reads. However, a majority of sequenced reads were still annotated to Neoptera, indicating that the host genome amplification was not completely inhibited. A similar approach was used in mosquitoes, with comparable success (Belda et al., 2017).

The eukaryotic microbiota included a wide variety of taxa outside of the previously characterized fungi associated with BSF. The taxonomic group Alveolata was found in the eukaryotic microbiota. This group includes Apicomplexa, a large parasitic group; Chloroplastida, a group of photosynthetic eukaryotes; Discoba, a major supergroup of unicellular eukaryotes, the Tetramitia family, most of which are bacterivorous and pathogenic, and the fungal kingdom, including the Trichosporonaceae family (De Jonckheere and Brown, 2005). The only Discoba annotated to the species level was the *Tetramitus entericus* genus, which includes human parasites. *Vermamoeba vermiformis*, a ubiquitous free-living amoeba of notable public health interest because it harbors other microorganisms and may protect against pathogenic bacteria and viruses, was also detected in the BSF microbiota (Siddiqui et al., 2021). Also found in the eukaryotic microbiota were *Apicomplexa sp*. and the Eugregarinorida order, common monoxenous parasites of many insects. They are transmitted by oral ingestion of oocysts and can also be transmitted through egg laying. Their progressive increase through BSF development of the stages (<0.01% at A-X0 and EGG, 0.07% EL, 0.15% LL, 0.13% PP, and 1.87% P) could reflect the parasite multiplication following neonates hatching, and their reduced abundance in imagoes could be attributed to starvation at this stage or the result of holometabolous processes.

Most taxa identified at the species level were Fungi. The most prevalent member was *Pichia*, which was also the only strict core microbiota taxon in the present study. *Pichia* was also the most abundant genus found in association with the Gainesville-reared BSF in a previous study on fungal microbiota (Varotto Boccazzi et al., 2017). The emerging nosocomial *Wickerhamomyces anomalus* was also identified (Zhang L. et al., 2021). A complete list of all the eukaryote taxa identified to the species level can be found in the Supplementary material.

Rare taxa seemed to play an important role in evenness diversity between gut regions, but this may have been exacerbated by the limited detection ability of eukaryotes other than the host. As expected, many taxa were unassigned. Eukaryotic microorganisms have not been investigated as intensively as bacteria and most of the species present in databases were identified because of their relevance to health and pathologies. Therefore, a notable bias exists toward potentially pathogenic eukaryotic microorganisms, which explains why most of the 18S rDNA sequences from this study matched pathogenic taxa. While bacterial microbiota investigations are routinely performed, inquiries into eukaryotic microbiota outside of fungal communities are scarce. Many practical challenges still exist, and more attention should be given to tackling these challenges.

## 5. Conclusion

In conclusion, this study aimed to explore the factors influencing the structure of the BSF microbiota throughout its complete life cycle. We also investigated the composition and dynamics of eukaryotic members of the microbiota during the developmental stages of BSF. While bacterial microbiota analysis is common in microbiological laboratories, research on non-fungal eukaryotic microbiota remains limited. Addressing the practical challenges of studying eukaryotic microbiota is crucial, as their significance in host systems remains poorly understood.

Our findings suggest that the holometabolous process plays a key role in establishing the microbiota. Despite the common practice of transferring BSF larvae to new substrates at the beginning of the larval cycle, our study indicates that the microbiota inherited from parents (recruited from the egg surface) contributes significantly to substrate adaptation and microbiota stability. We propose that larvae transferred to a new substrate experience adaptive dysbiosis, recruiting a new microbiota optimized for the available resources. Therefore, transferring BSF to a new substrate at the pre-pupae stage may facilitate and expedite adaptation to the new environment. Furthermore, our results provide valuable insights for the selection and development of a synthetic microbiota assembly for further exploration of the roles and relationships of microorganisms in BSF development, health, and competences.

To better understand the functional aspects of the rearing substrate-associated microbiota, a comprehensive investigation is needed to determine the extent to which the microbiota is recruited based on its functional capacity to exploit the nutritional resources of the substrate. This could help explain the variations in taxonomic composition across studies while ensuring a similar functional repertoire. While the taxonomic composition of the microbiota may differ, they may share functional traits that are advantageous for BSF, triggered by the substrate composition.

Therefore, in this study, we identified several factors influencing the selective recruitment and assembly of the microbiota, including the presence of microorganisms on the egg or substrate (heritability and availability), the functional competence of the microorganisms (e.g., the ability to make resources accessible to larvae), substrate adaptation (metagenomic plasticity), the developmental stage of the host, and functional gut regions of the host.

## Data availability statement

The data presented in the study are deposited in the NCBI BioProject Database Sequence Read Archive repository, accession number PRJNA971599 (https://www.ncbi.nlm.nih.gov/sra/PRJNA971599).

## Author contributions

ND, GV, and LA designed the experiment. LA performed the experiment's manipulations, computational analysis, data interpretation, and wrote the manuscript. GV and ND conducted the fund acquisition. ND, GV, and M-HD reviewed and edited the manuscript. All authors have read and approved the manuscript.
