# Supplementary material for: Microbiota is structured by gut regions, life stage, and diet in the Black Soldier Fly (Hermetia illucens)
Source: Front Microbiol. 2023 Aug 17;14:1221728. doi: 10.3389/fmicb.2023.1221728 (PMC10469785; doi:10.3389/fmicb.2023.1221728)
Supplement: Supplementary file 1 [file Data_Sheet_1.docx]

Supplementary Material

Microbiota is structured by gut regions, life stage and diet in Black Soldier Fly (*Hermetia illucens*)

Laurence Auger^*^, Marie-Hélène Deschamps, Grant Vandenberg, Nicolas Derome

*** Correspondence:** Corresponding Author: laurence.auger.1@ulaval.ca

# Supplementary Figures and Tables

## Supplementary Figures


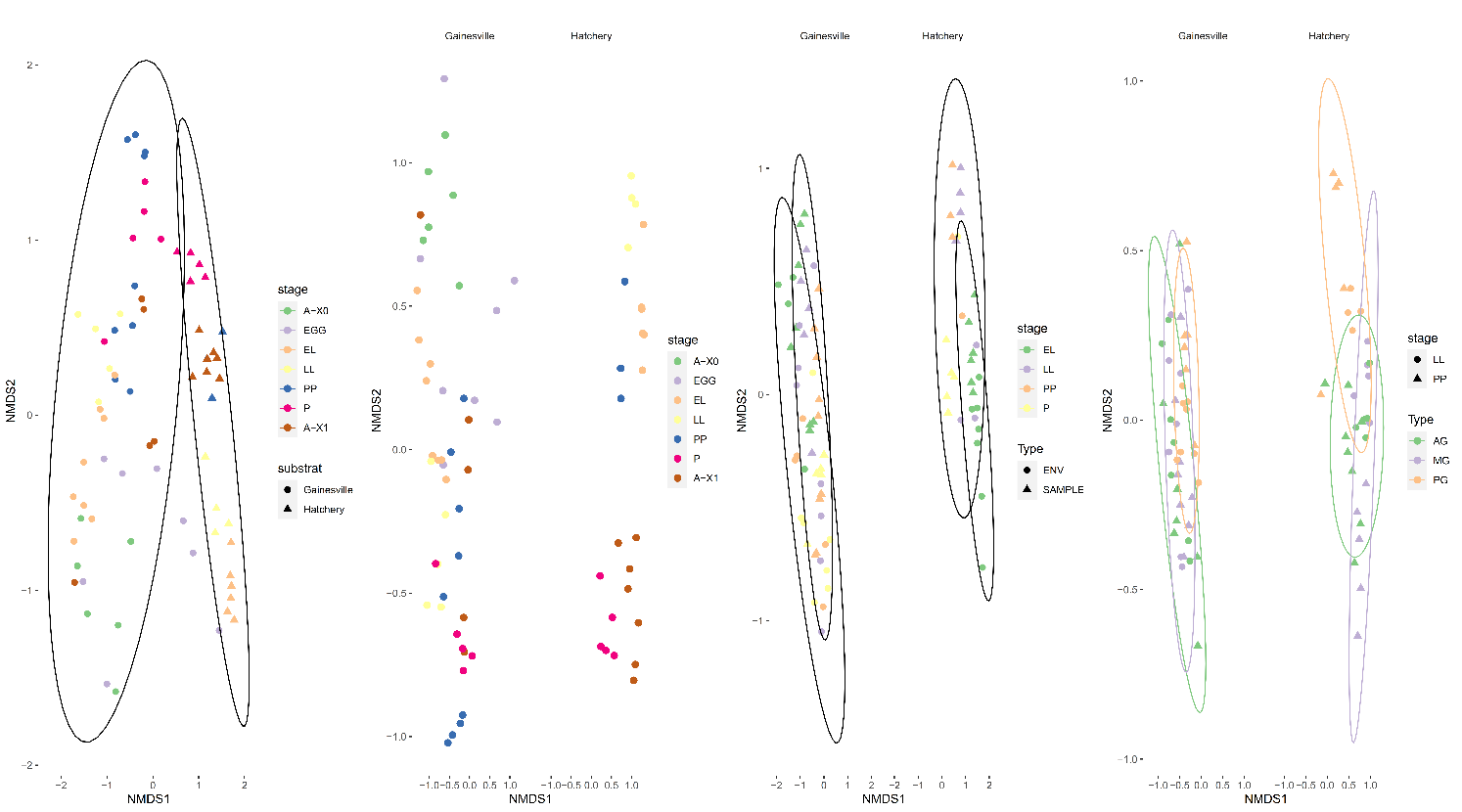

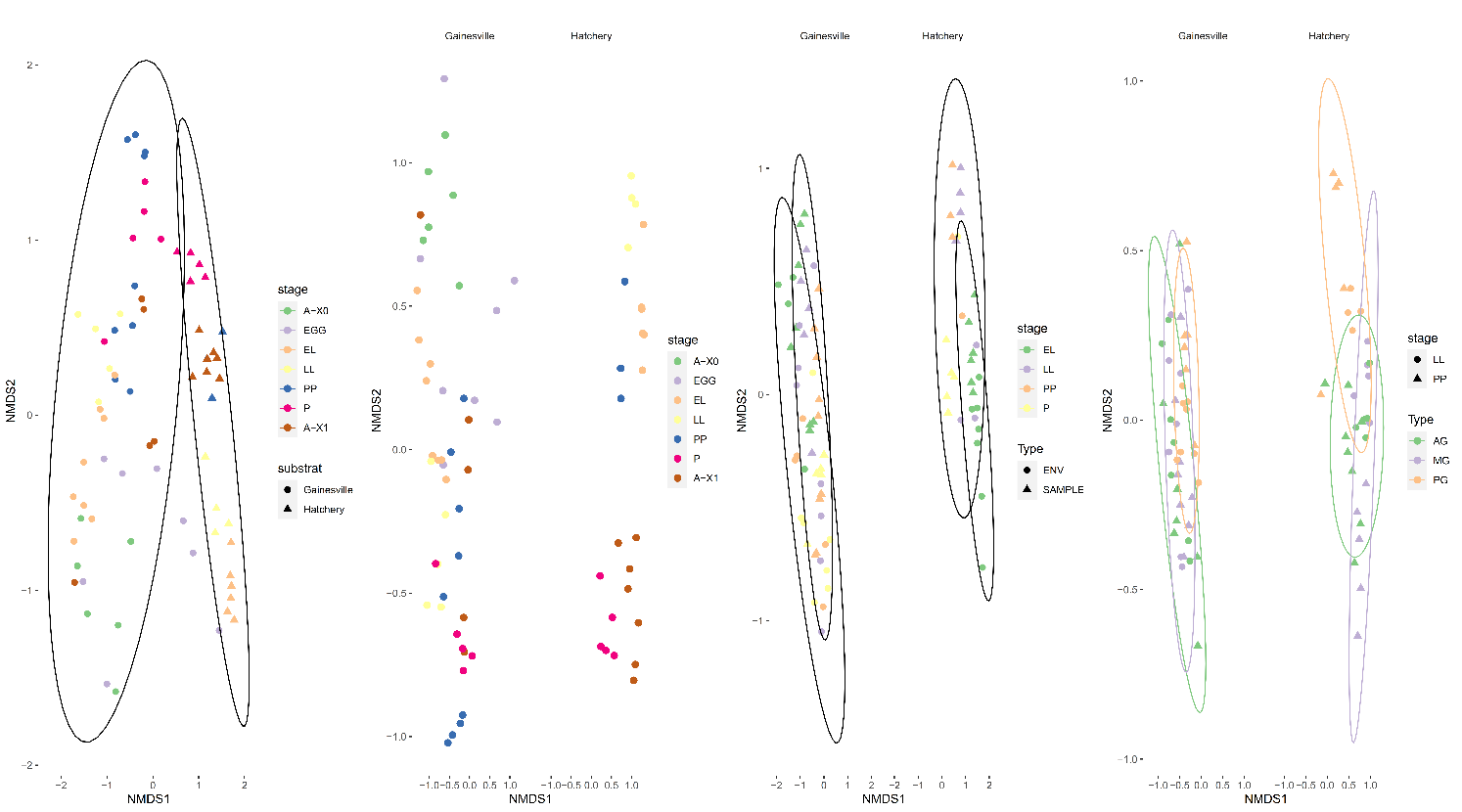


**Supplementary Figure 1. Clustering of the bacterial microbiota in NMDS ordinations plots.** The bacterial composition is clustered in NMDS ordination plots based on Bray-Curtis distances. Clustering is shown by rearing substrate (first column), by life stage (second column), by sample type: substrate or whole BSF (third column).


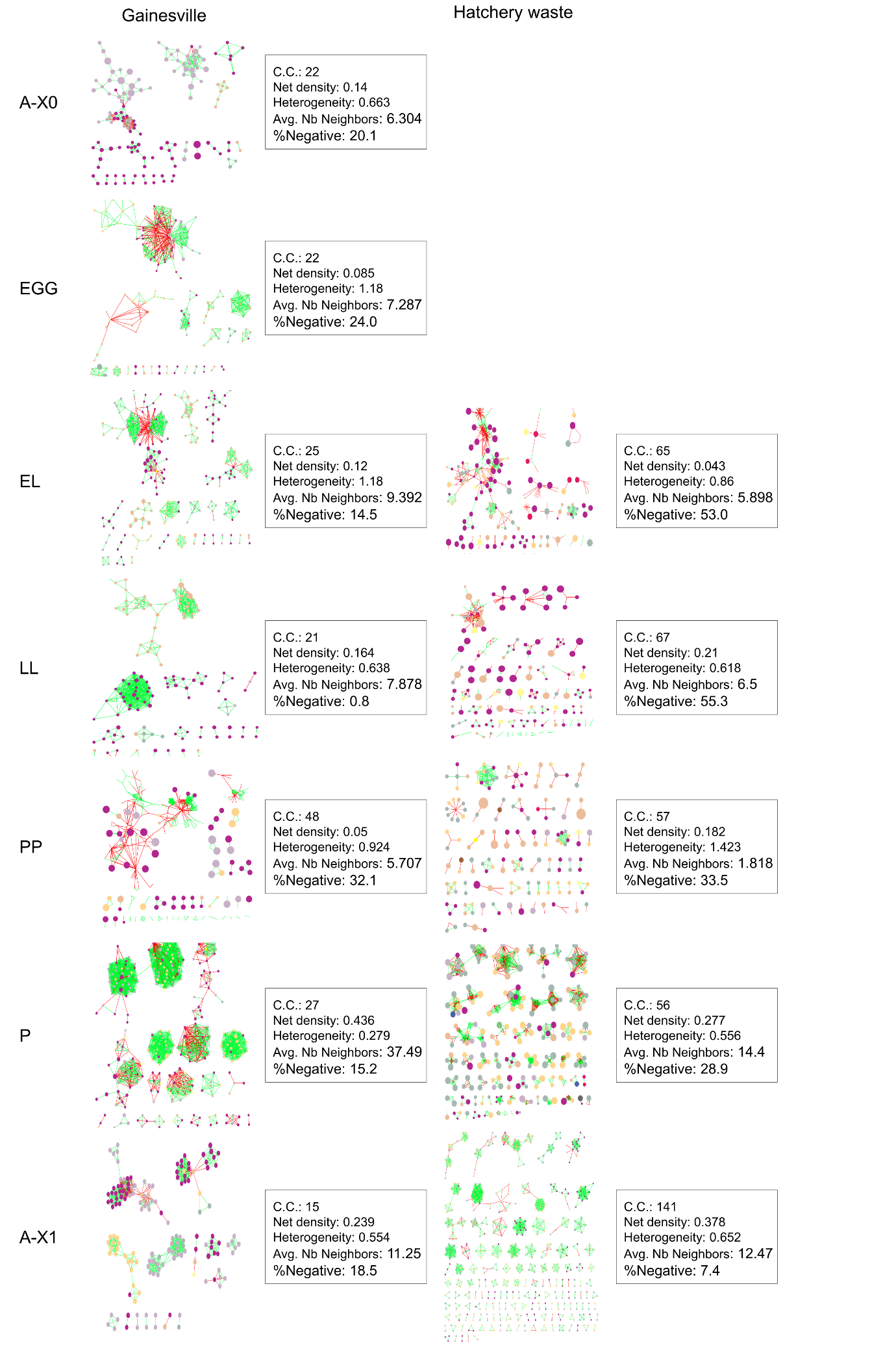


**Supplementary Figure 2.** **Interactions networks and parameters of whole BSF microbiota.** Edges represent significant interactions; red edges illustrate co-exclusion while green edges indicate co-occurrence. Percentage of negative interactions (negative edges/total number of edges*100) is indicated for each network. Each node is a unique ASV, node size is proportional to the relative abundance of each ASV in the sample group. Labels are the taxonomic family of the corresponding node, and font size is proportional to the number of connected edges (interactions). (A) A-X0; (B) EGG; (C) EL; (D) LL; (E) PP; (F) P; (G) A-X1.


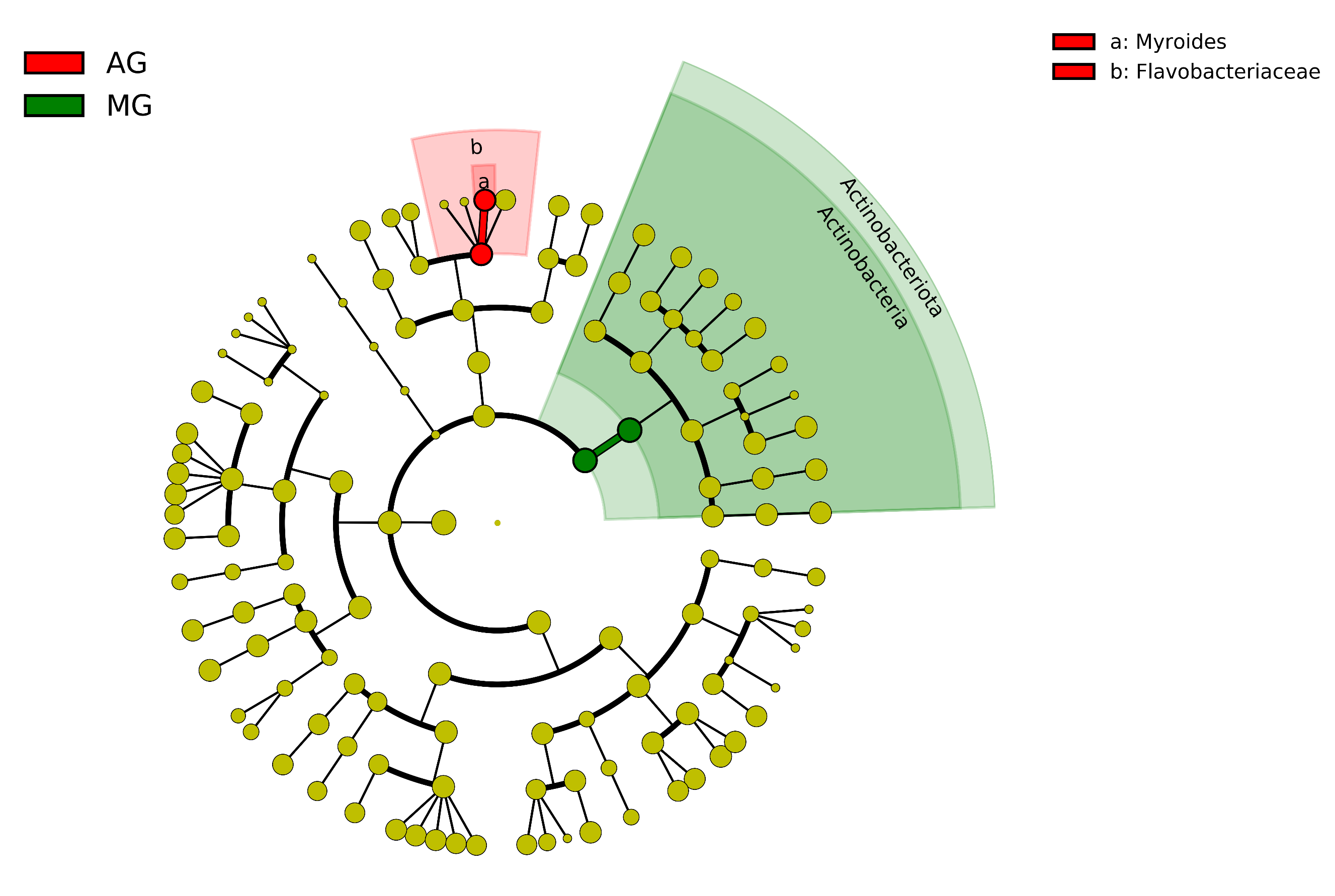


Gainesville

Hatchery waste


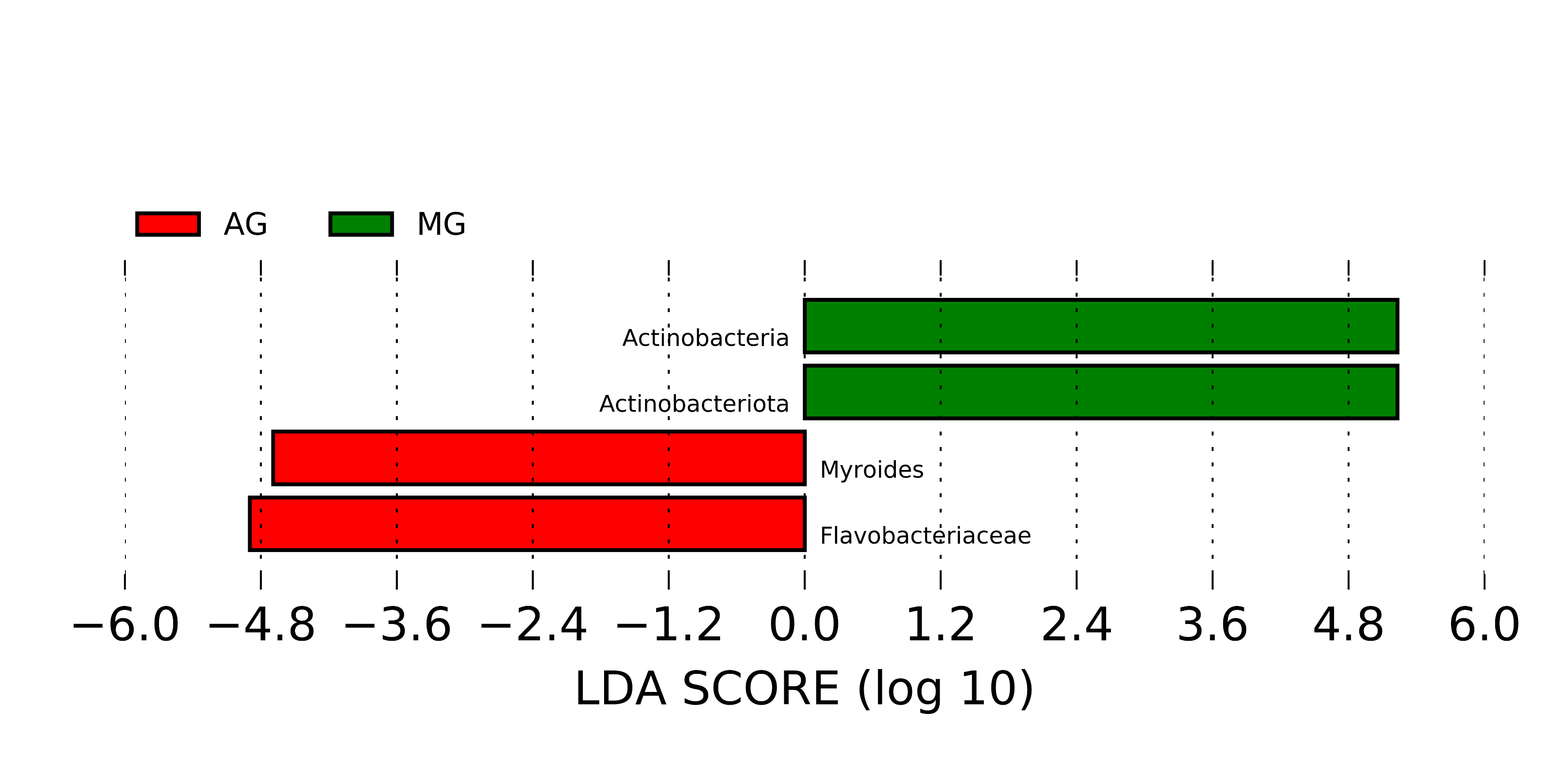

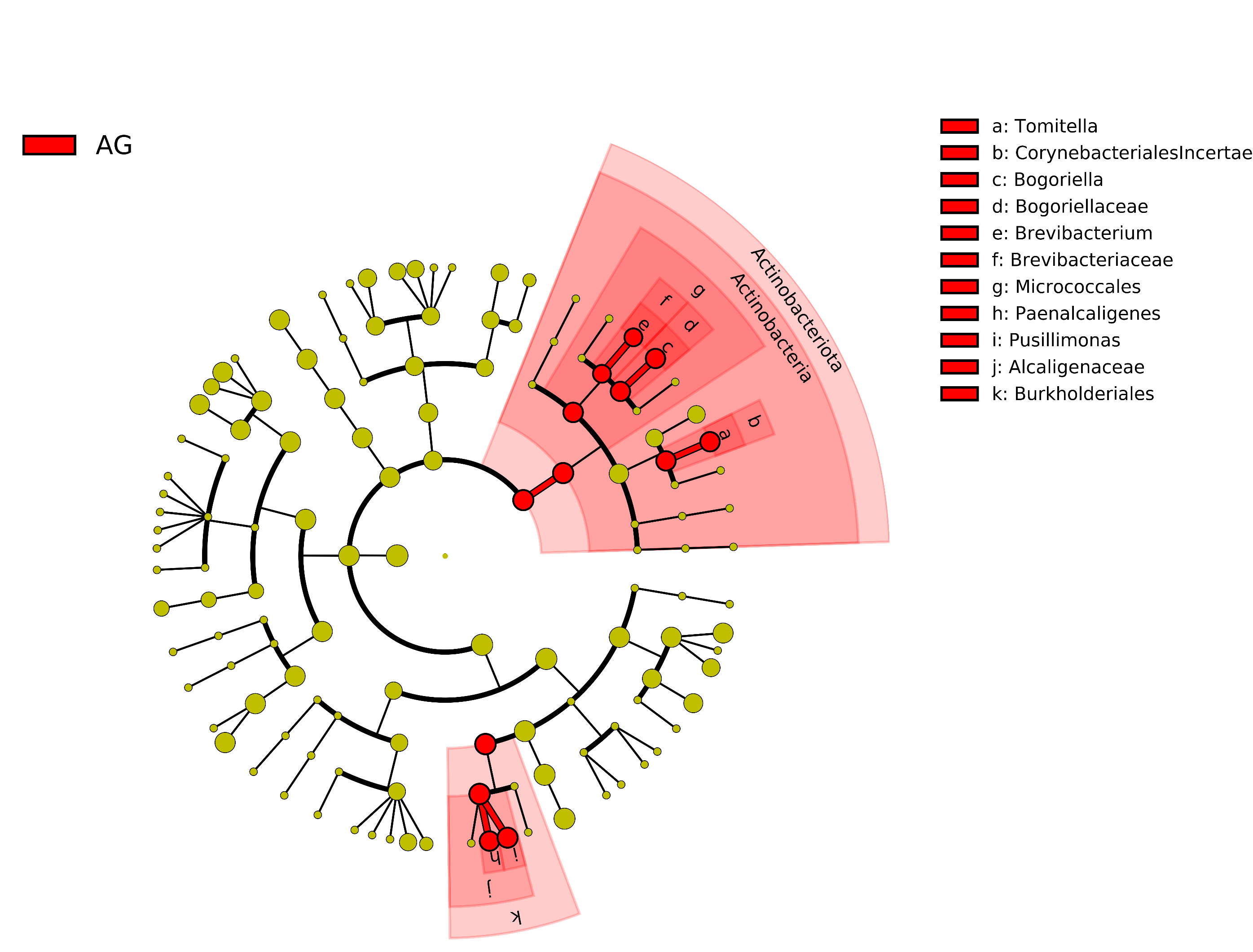

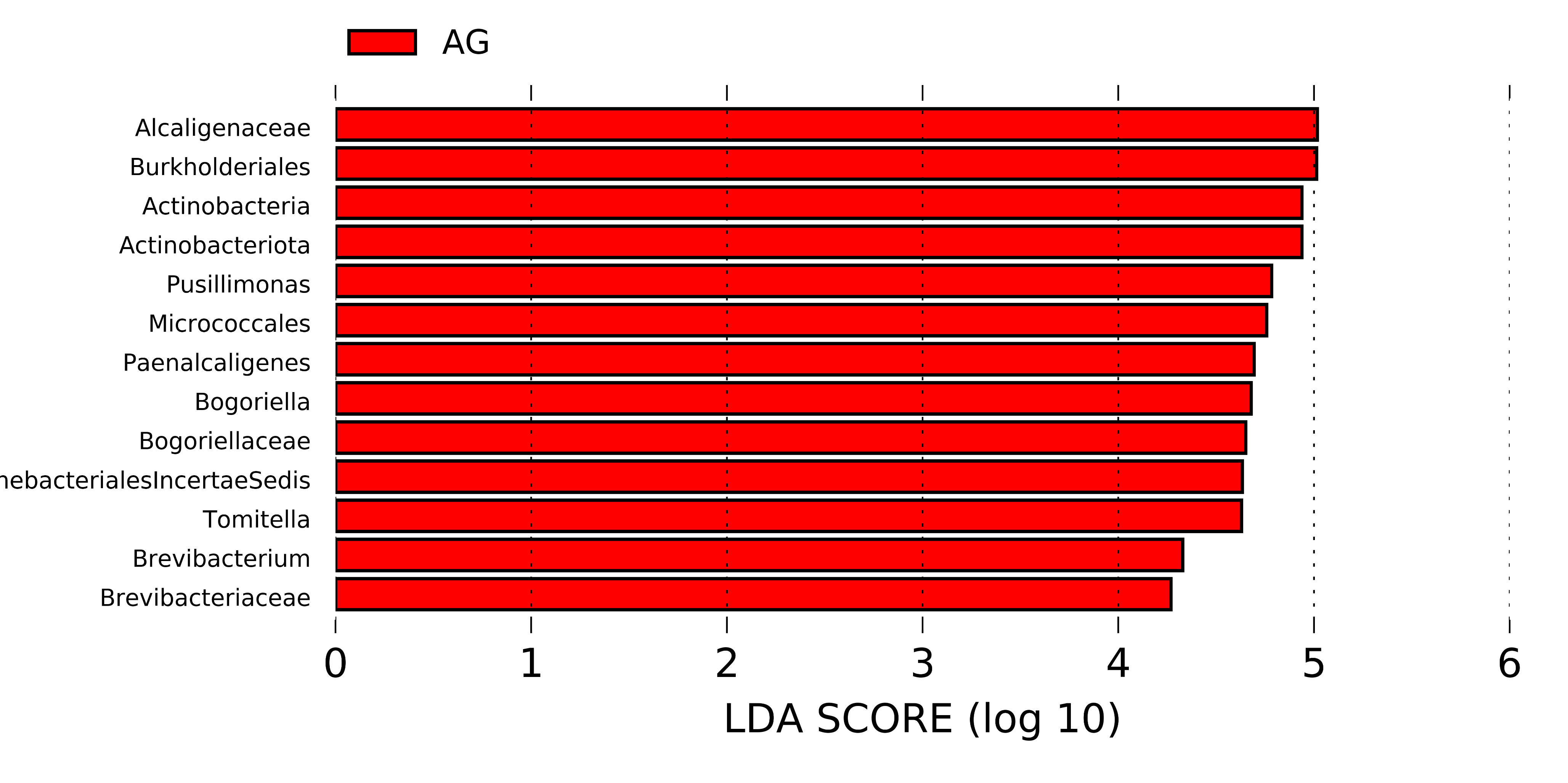

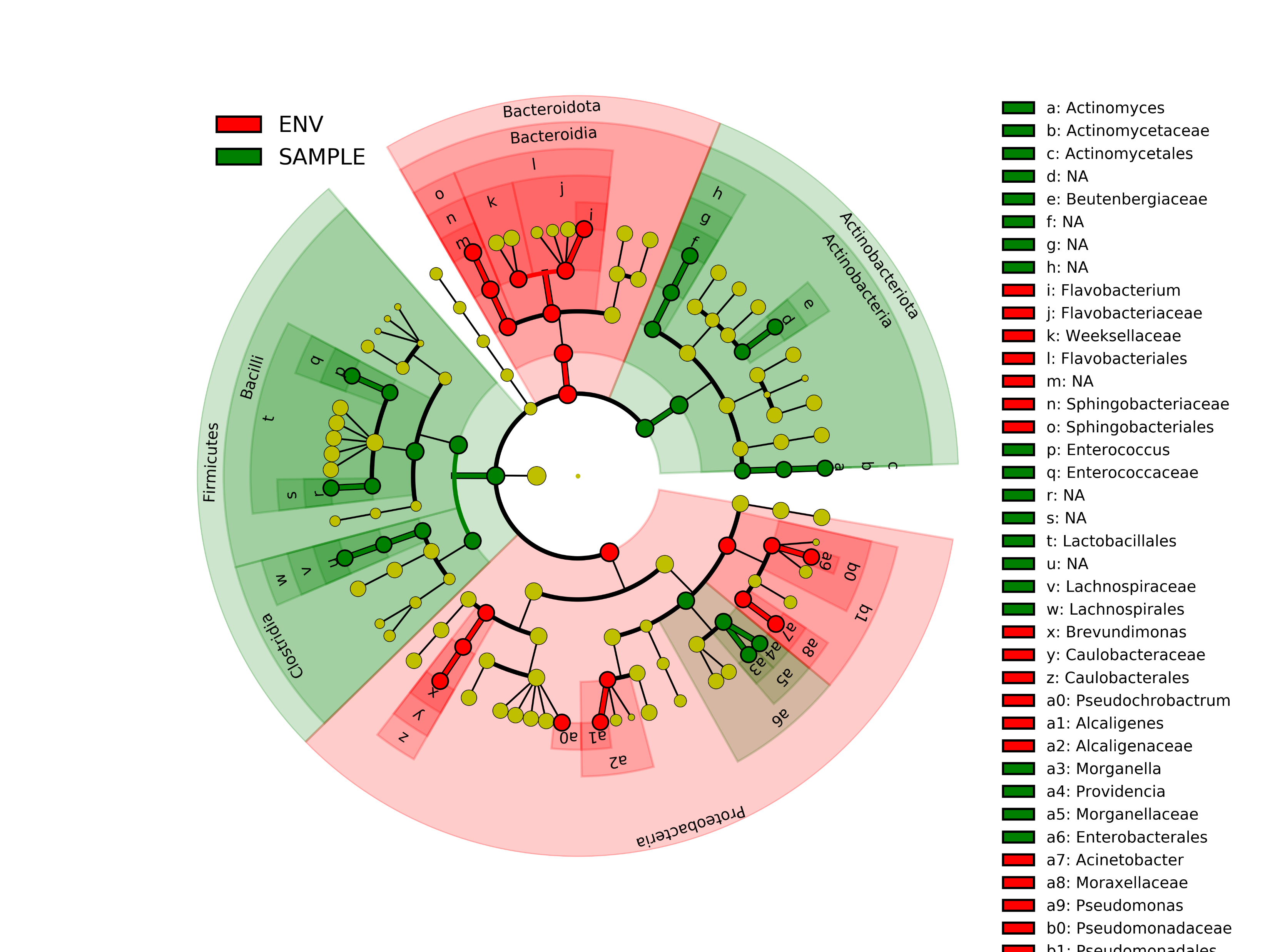

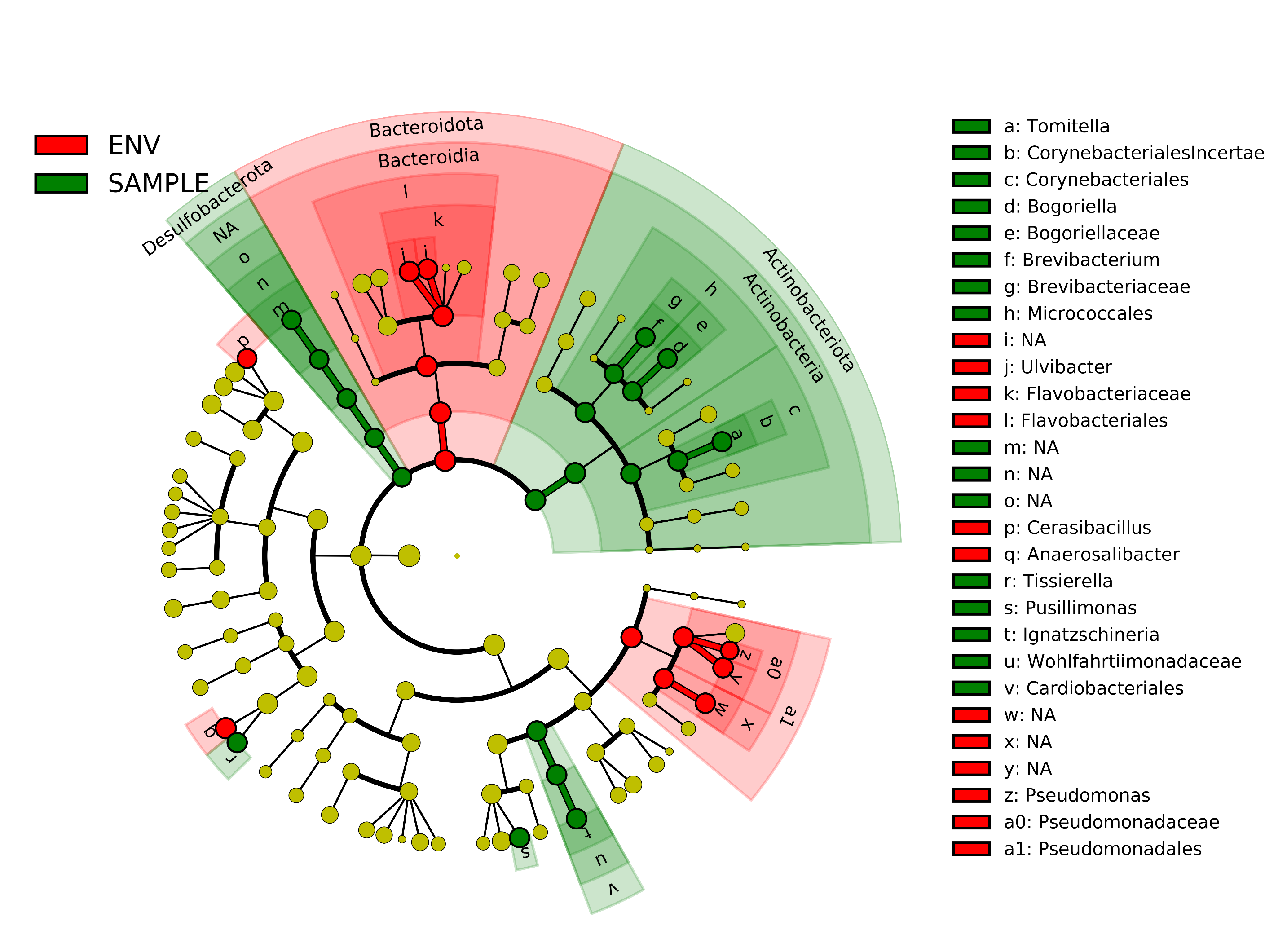

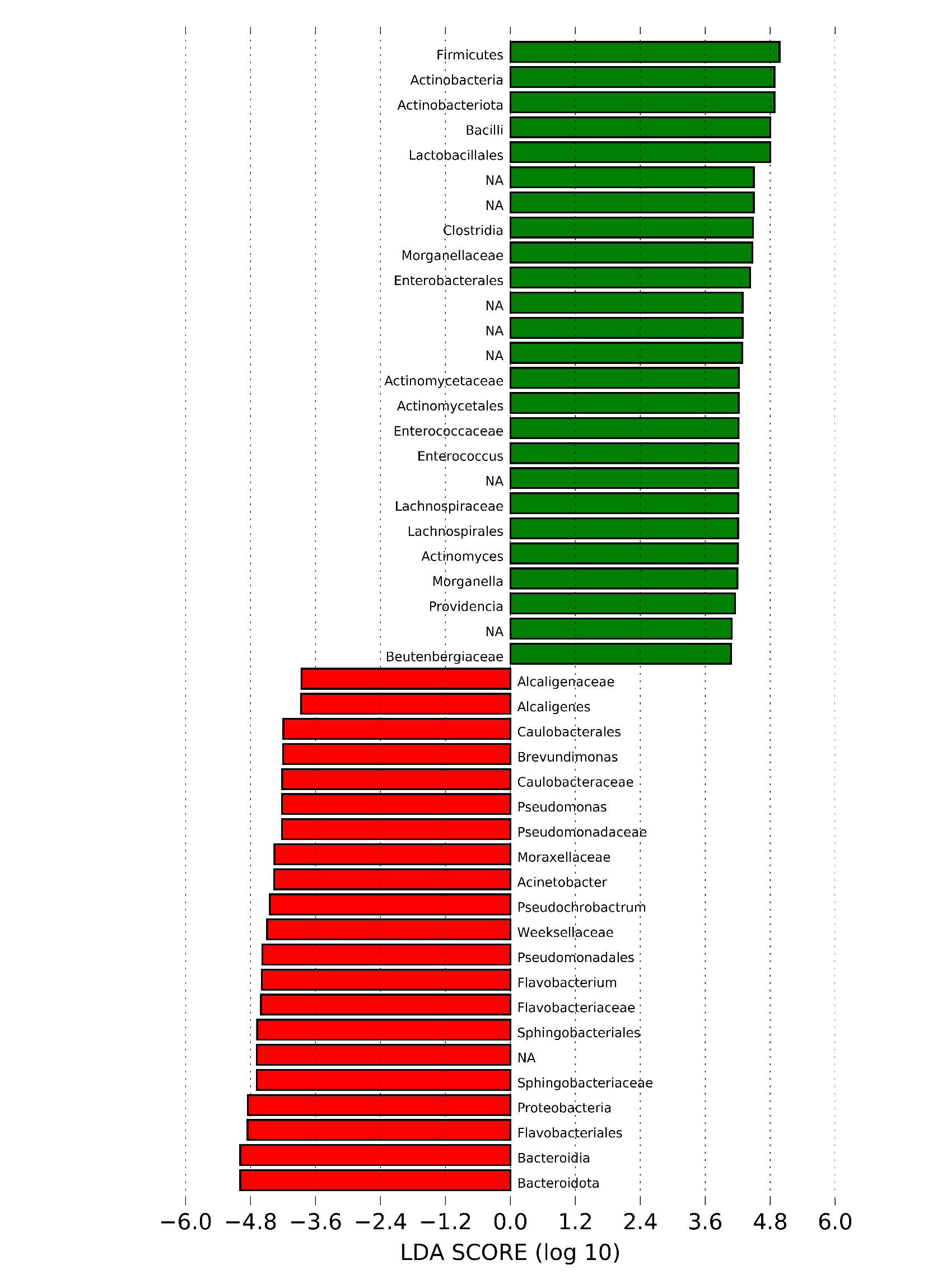

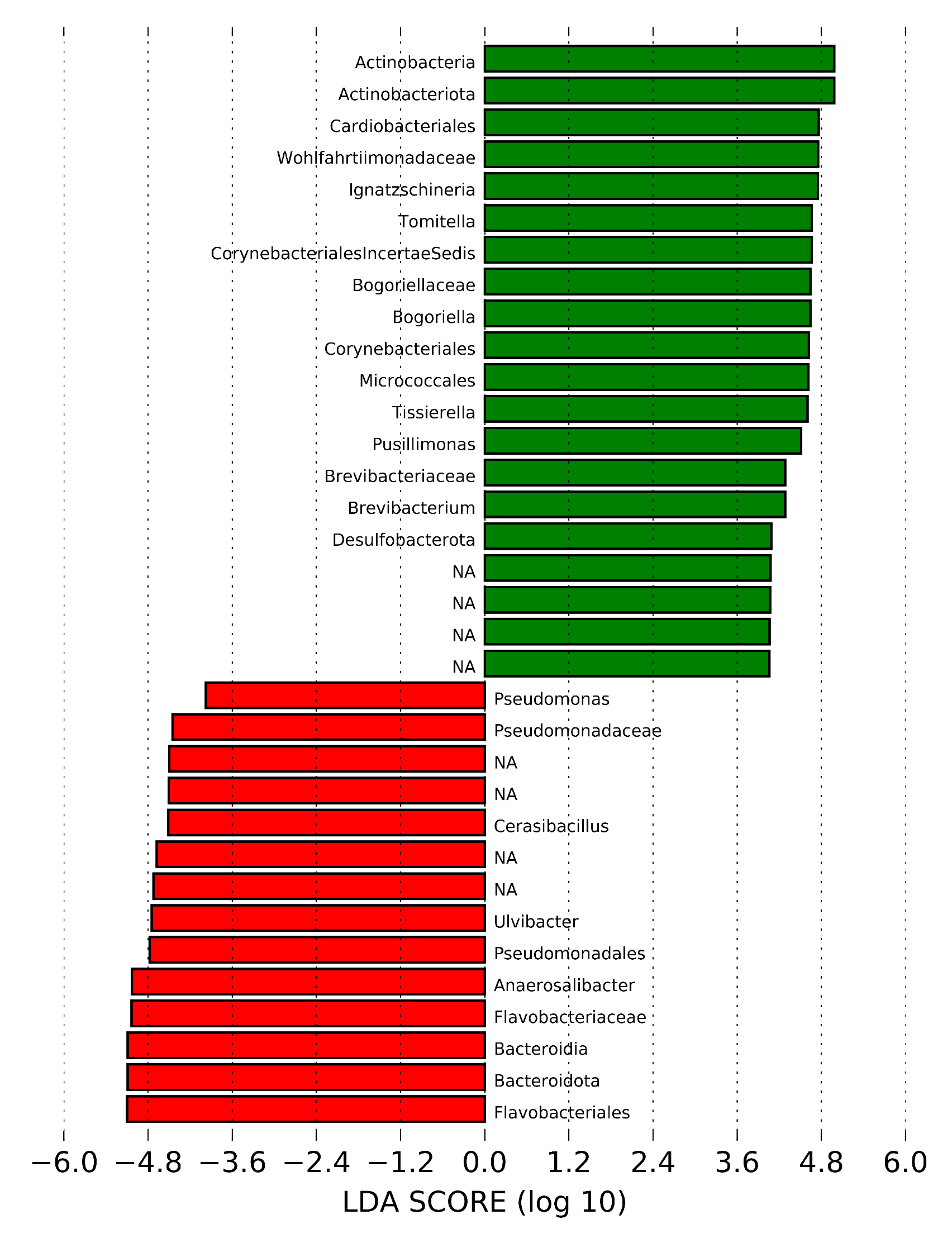

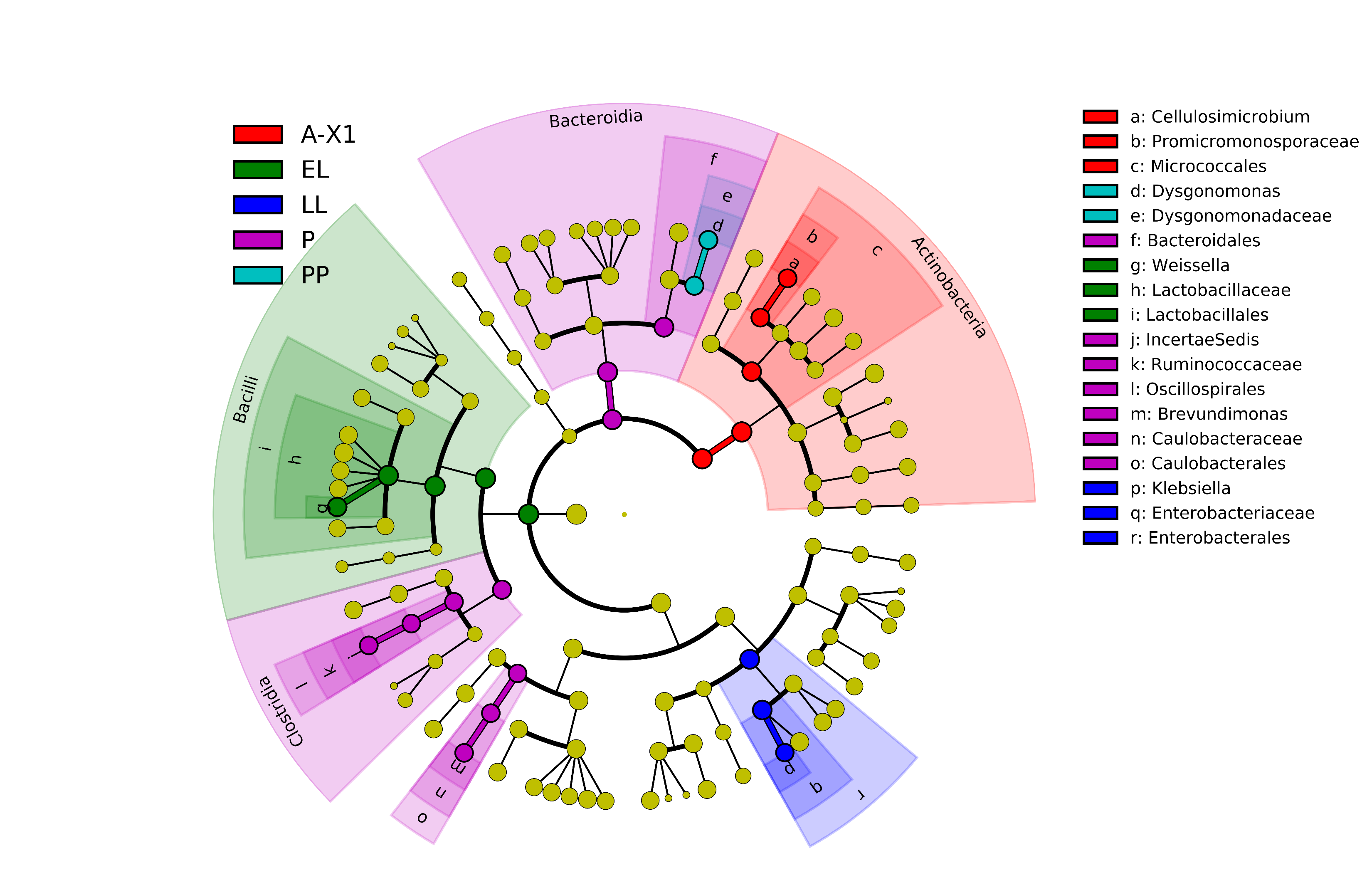

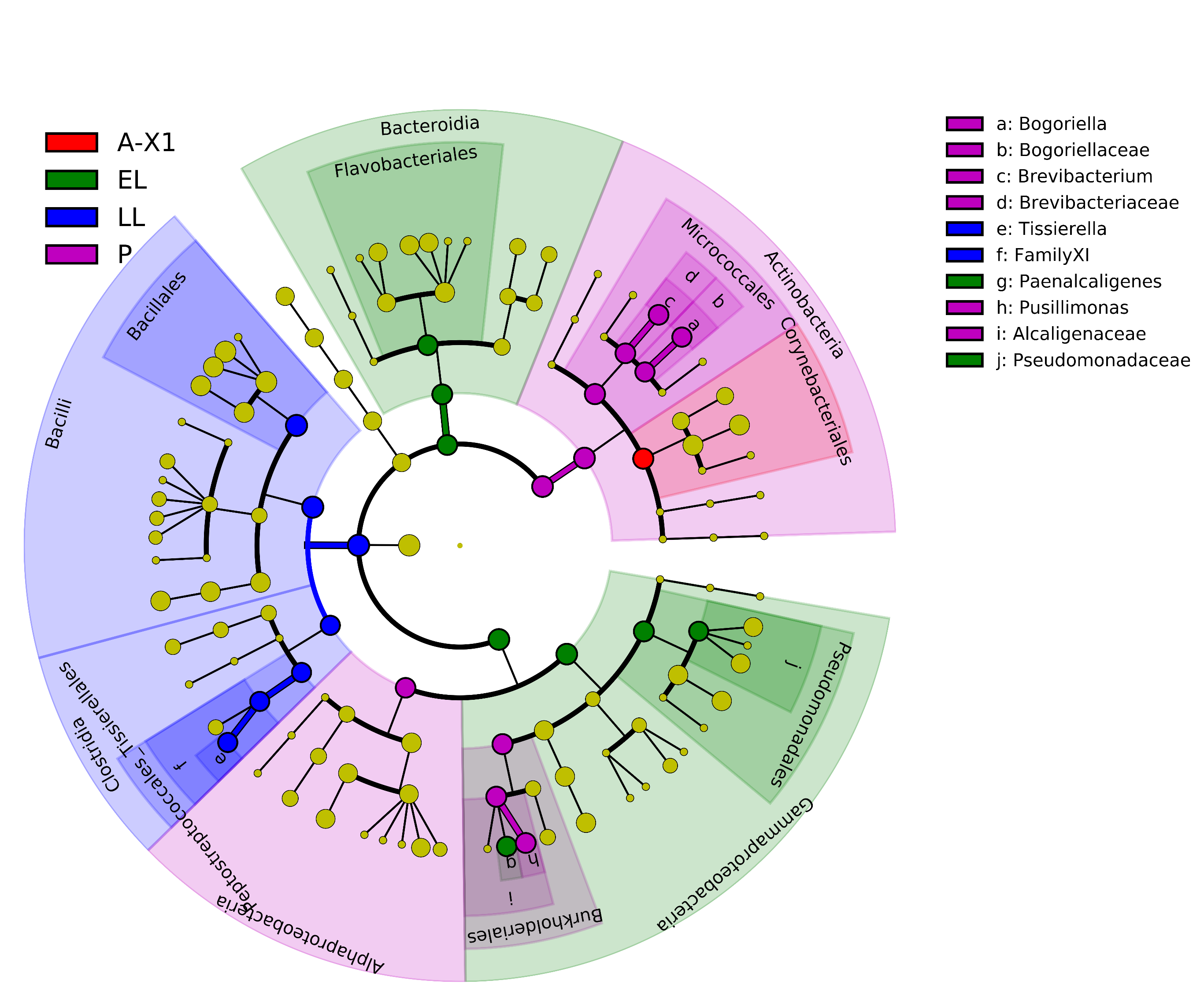

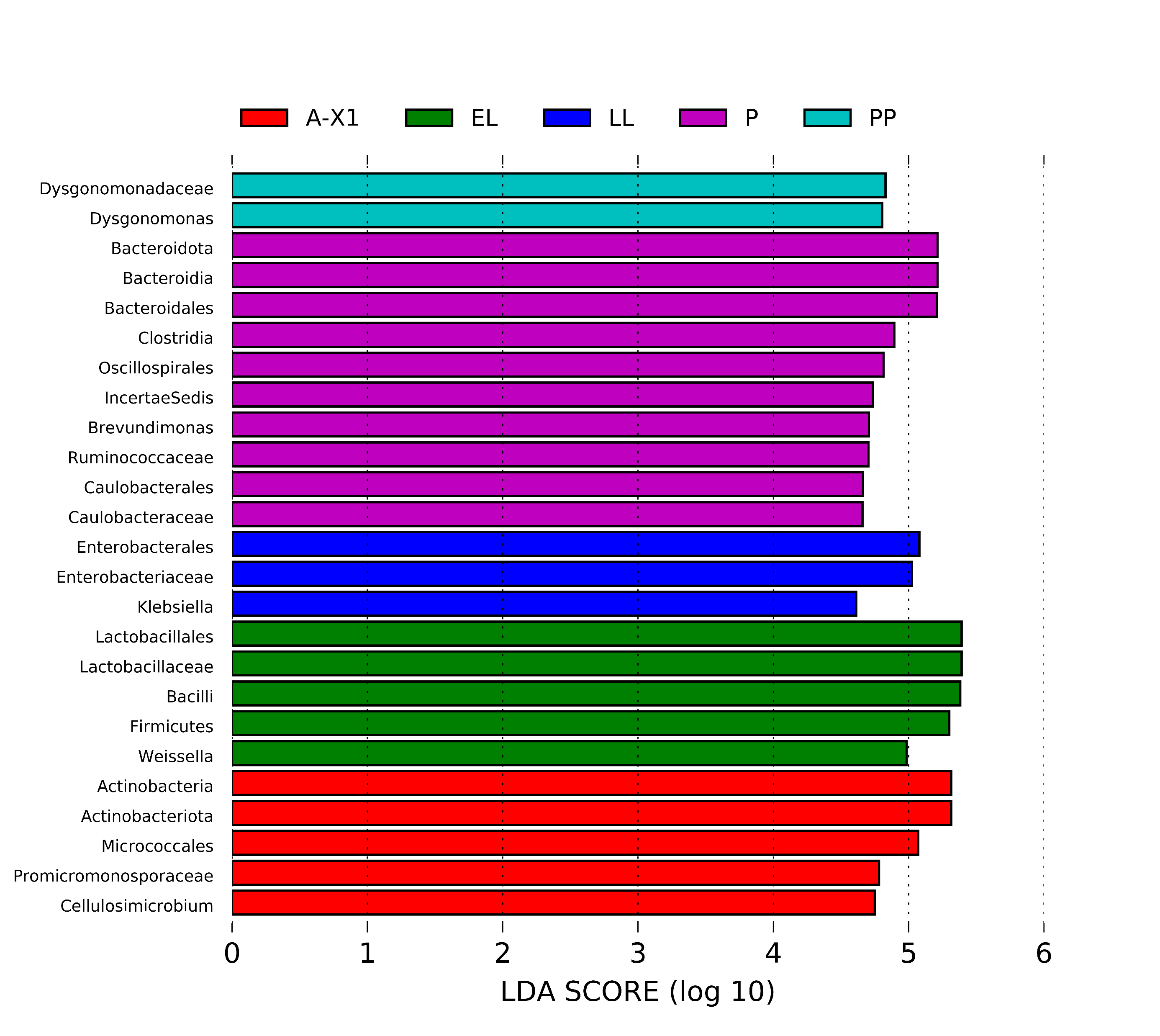

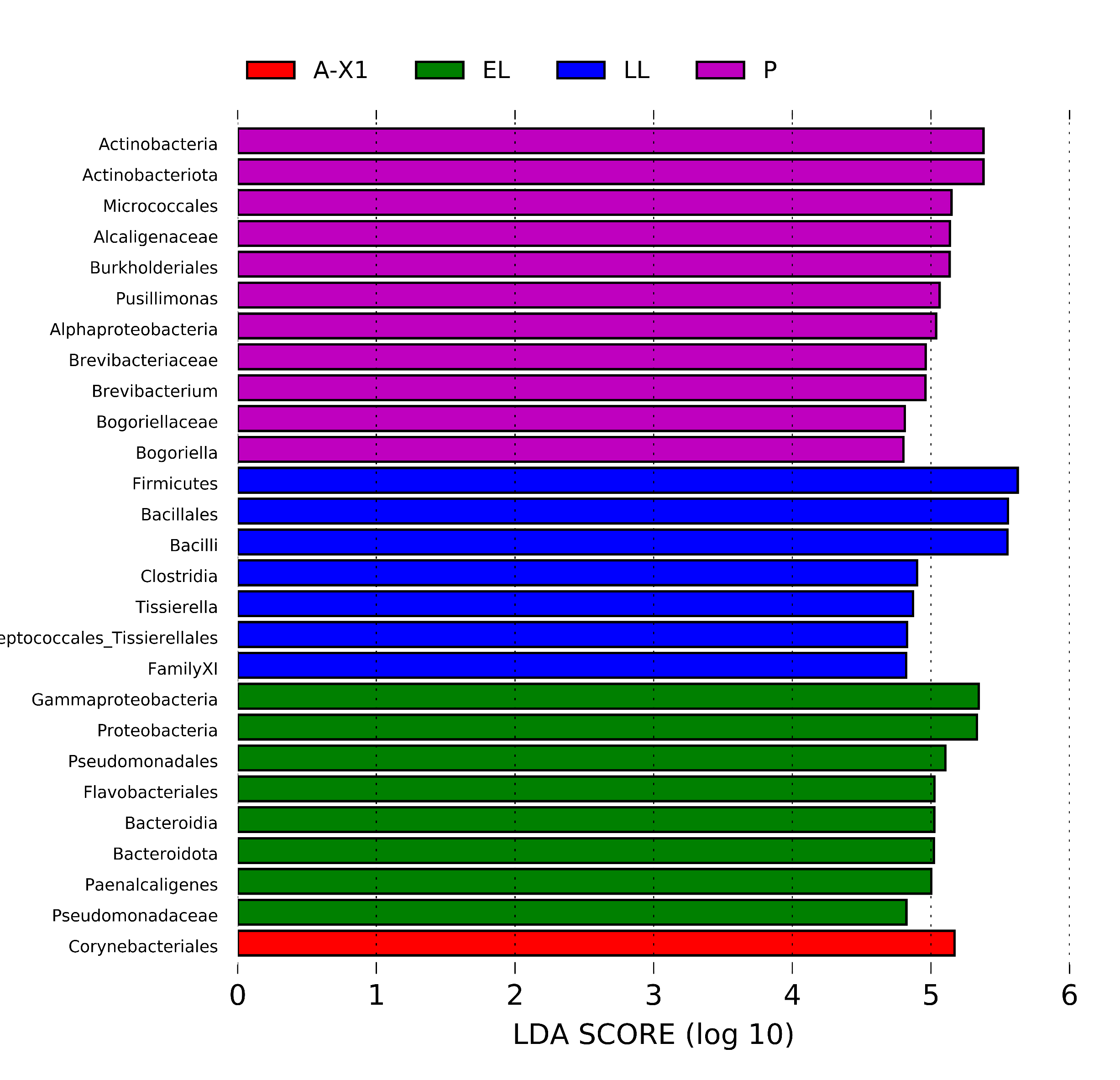


**Supplementary Figure 3. LEfSe analysis of the discriminant bacterial taxa between groups.** First row: Cladogram of the phylogenetic distribution of the bacterial taxa discriminant (LDA > 4) for the whole BSF (green) and the substrate (red), with adjacent histogram of the taxa and their LDA score. Second row: Cladogram of the phylogenetic distribution of the bacterial taxa associated to BSF discriminant for each developmental stages: in red A-X1, in green EL, in blue LL, in purple P and in turquoise PP, with corresponding histogram presenting the LDA score of the taxa. Third row: Cladogram of the phylogenetic distribution of the bacterial taxa discriminant for each gut regions of the BSF, in red AG and in green MG, with corresponding histogram presenting the LDA score of the taxa.

## Supplementary Tables

| ASV_ID | Kingdom | Phylum | Class | Order | Family | Genus | Species | AC |
| --- | --- | --- | --- | --- | --- | --- | --- | --- |
| ASV509 | Eukaryota | SAR | Alveolata | Apicomplexa | Gregarinasina | Eugregarinorida | Apicomplexa_sp._1_KCW-2013 | KC890798.1.1780 |
| ASV61 | Eukaryota | Opisthokonta | Nucletmycea | Fungi | Aspergillaceae | Aspergillus | Aspergillus_sp._Y6-1 | KP872533.1.1733 |
| ASV197 | Eukaryota | Opisthokonta | Nucletmycea | Fungi | Aspergillaceae | Aspergillus | Aspergillus_terreus | NA |
| ASV648 | Eukaryota | Opisthokonta | Nucletmycea | Fungi | Incertae_Sedis | Candida | Candida_sp._JCM_15000 | BCKB01000007.4153.5896 |
| ASV395 | Eukaryota | Opisthokonta | Nucletmycea | Fungi | Debaryomycetaceae | Candida-Lodderomyces_clade | Candida_tropicalis_MYA-3404 | NA |
| ASV40 | Eukaryota | Opisthokonta | Nucletmycea | Fungi | Phaffomycetaceae | Cyberlindnera-Candida_clade | Cyberlindnera_fabianii | NA |
| ASV11 | Eukaryota | Opisthokonta | Nucletmycea | Fungi | Incertae_Sedis | Candida | Diutina_mesorugosa | KY464166.1.2622 |
| ASV308 | Eukaryota | SAR | Alveolata | Apicomplexa | Eugregarinorida | Gregarina | Heterocapsaceae_environmental_sample | EF024723.1.1773 |
| ASV120 | Eukaryota | Opisthokonta | Nucletmycea | Fungi | Malasseziaceae | Malassezia | Malassezia_globosa_CBS_7966 | AAYY01000016.137081.138848 |
| ASV432 | Eukaryota | Opisthokonta | Nucletmycea | Fungi | Mucoraceae | Mucor | Mucor_sp._BMC2 | KC009579.1.1808 |
| ASV171 | Eukaryota | Opisthokonta | Nucletmycea | Fungi | Aspergillaceae | Penicillium | Penicillium_capsulatum | NA |
| ASV163 | Eukaryota | Opisthokonta | Nucletmycea | Fungi | Aspergillaceae | Penicillium | Penicillium_chrysogenum | M55628.1.1797 |
| ASV190 | Eukaryota | SAR | Stramenopiles | Peronosporomycetes | Phytophthora | Phytophthora_nicotianae | Phytophthora_nicotianae | NA |
| ASV1 | Eukaryota | Opisthokonta | Nucletmycea | Fungi | Pichiaceae | Pichia | Pichia_kudriavzevii | JF274497.1.1675 |
| ASV21 | Eukaryota | Opisthokonta | Nucletmycea | Fungi | Saccharomycetaceae | Saccharomyces | Saccharomyces_cerevisiae_W303 | NA |
| ASV321 | Eukaryota | Excavata | Discoba | Discicristata | Tetramitia | Learamoeba | Tetramitus_entericus | AJ224889.1.2106 |
| ASV19 | Eukaryota | Opisthokonta | Nucletmycea | Fungi | Trichosporonaceae | Trichosporon | Trichosporon_asahii_var._asahii_CBS_2479 | ALBS01000282.5578.7112 |
| ASV66 | Eukaryota | Opisthokonta | Nucletmycea | Fungi | Incertae_Sedis | Candida | uncultured_Candida | JX132057.1.1745 |
| ASV4 | Eukaryota | Opisthokonta | Nucletmycea | Fungi | Dipodascaceae | Geotrichum | uncultured_eukaryote | AB902190.1.1779 |
| ASV236 | Eukaryota | Opisthokonta | Nucletmycea | Fungi | Malasseziaceae | Malassezia | uncultured_fungus | NA |
| ASV414 | Eukaryota | Opisthokonta | Nucletmycea | Fungi | uncultured | uncultured_Tremellaceae | uncultured_Tremellaceae | NA |
| ASV305 | Eukaryota | Opisthokonta | Nucletmycea | Fungi | Aspergillaceae | Aspergillus | unidentified | LG070291.1.1740 |
| ASV536 | Eukaryota | Amoebozoa | Tubulinea | Arcellinida | Echinamoebida | Vermamoeba | Vermamoeba_vermiformis | AF426157.1.1841 |
| ASV36 | Eukaryota | Opisthokonta | Nucletmycea | Fungi | Phaffomycetaceae | Wickerhamomyces-Candida_clade | Wickerhamomyces_anomalus | NA |

**Supplementary Table 1. Unique species found in the eukaryotic microbiota.** Taxa identified to the specie level are presented in the table, with only the first ASVs annotated to this level presented for each unique species. Accession numbers for the SILVA database. NA indicates un-assigned taxonomic annotation for the rank.

| Substrate | Stage | Nodes | Edges | | Avg. neighbors | Clustering coef. | CC | Negative edges | Positive edges | Negative interactions (%) | CC normalized |
| --- | --- | --- | --- | --- | --- | --- | --- | --- | --- | --- | --- |
| Gainesville | A-X0 | 143 | | 309 | 6.304 | 0.498 | 22 | 62 | 247 | 20% | 0.153846 |
|  | EGG | 205 | | 584 | 7.287 | 0.635 | 22 | 140 | 444 | 24% | 0.107317 |
|  | EL | 235 | | 684 | 9.392 | 0.626 | 25 | 92 | 592 | 13% | 0.106383 |
|  | LL | 142 | | 526 | 7.878 | 0.653 | 21 | 4 | 522 | 1% | 0.147887 |
|  | PP | 270 | | 535 | 5.707 | 0.302 | 48 | 172 | 363 | 32% | 0.177778 |
|  | P | 311 | | 2799 | 37.494 | 0.952 | 27 | 425 | 2374 | 15% | 0.086817 |
|  | A-X1 | 158 | | 686 | 11.25 | 0.81 | 15 | 127 | 559 | 19% | 0.094937 |
| Hatchery | EL | 358 | | 610 | 5.898 | 0.501 | 65 | 323 | 287 | 53% | 0.181564 |
|  | LL | 253 | | 309 | 6.5 | 0.24 | 67 | 171 | 138 | 55% | 0.264822 |
|  | PP | 181 | | 215 | 1.818 | 0 | 57 | 72 | 143 | 33% | 0.314917 |
|  | P | 527 | | 2844 | 14.704 | 0.919 | 56 | 821 | 2023 | 29% | 0.106262 |
|  | A-X1 | 667 | | 1696 | 12.471 | 0.746 | 141 | 125 | 1571 | 7% | 0.211394 |

**Supplementary Table 2.** Report of the whole BSF microbiota interactions networks parameters analyzed with Cytoscape as undirected graph. Nodes represent a unique ASV, and edges are interactions. The Average number of neighbors (Avg. neighbors) is the average connectivity of the nodes in the network. Clustering coefficient is an average of the ratio of the number of edges between neighbors by the maximum number of edges possible for all nodes. Connected components (CC) are the number of groups of connected components (nodes). Negative edges are negative interactions and positive edges are positive interactions. The ratio of negative interactions by the total number of interactions (edges) in the network are presented in percentage. In the last column, the number of connected components is normalized by the number of nodes (0 = all nodes are connected, 1 = all nodes are disconnected).
